# Tgfβ signaling stimulates glycolysis to promote the genesis of synovial joint interzone in developing mouse embryonic limbs

**DOI:** 10.1126/sciadv.adq4991

**Published:** 2025-01-08

**Authors:** Chao Song, Jasmin Koehnken Sawall, Xing Ji, Fangfang Song, Xueyang Liao, Renpeng Peng, Hao Ren, Eiki Koyama, Maurizio Pacifici, Fanxin Long

**Affiliations:** ^1^ Translational Research Program in Pediatric Orthopedics, Department of Surgery, The Children’s Hospital of Philadelphia, Philadelphia, PA, USA.; ^2^ Department of Orthopedic Surgery, Tongji Hospital, Huazhong University of Science and Technology, Wuhan, China.; ^3^ Deaprtment of Orthopedic Surgery, University of Pennsylvania, Philadelphia, PA, USA.

## Abstract

The initial interzone cells for synovial joints originate from chondrocytes, but such critical transition is minimally understood. With single-cell RNA sequencing (scRNA-seq) of murine embryonic knee joint primordia, we discovered that heightened expression of glycolysis genes characterized developing interzone cells when compared to flanking chondrocytes. Conditional deletion of the glucose transporters *Glut1* and/or *Glut3*, in either the incipient pre-skeletal mesenchyme with *Prx1Cre* or in chondrocytes with *Col2Cre*, disrupted interzone formation dose-dependently. In contrast, deletion of *Glut1*/*3* in established interzone cells with *Gdf5Cre* did not have similar severe disruption of joint development. scRNA-seq revealed that *Glut1/3* deletion by *Prx1Cre* impeded Tgfβ signaling in the developing interzone cells. Direct elimination of Tgfβ signaling with *Prx1Cre* partially phenocopied the deletion of *Glut1/3* in impairing interzone formation. Tgfβ stimulated glycolysis in chondrocytes via activation of mTOR and Hif1α in vitro. The data support that the essential conversion of chondrocytes to interzone cells requires a transient elevation of glycolysis partly dependent on Tgfβ signaling.

## Teaser

Stimulation of glycolysis by Tgfβ permits transition of chondrocytes to initial interzone cells during early joint development.

## INTRODUCTION

Synovial joint development in the embryonic limbs becomes appreciable with the emergence of a mesenchymal interzone at each prescribed joint site (*1*). Previous studies in mouse and chick embryos showed that the initial primordial skeletal template in early limbs is entirely cartilaginous without joints, and that the interzone originates from chondrocytes occupying each predetermined joint site that cease expression of cartilage markers such as *Col2a1* (*2*, *3*). The initial interzone not only marks the anatomical position of future joints, but also supplies progenitor cells for various joint tissues including articular cartilage, synovium, menisci, and cruciate ligaments in the knee (*4*). Following the initial interzone cells derived from chondrocytes, additional *Gdf5*-expressing cells are subsequently recruited from *Sox9*-positive progenitors surrounding the joint site and also contribute to distinct joint tissues over time (*5*, *6*). However, the mechanisms controlling the derivation of initial interzone cells from chondrocytes remain incompletely defined.

Extensive studies have implicated transforming growth factor–β (Tgfβ)/Bmp signaling in early joint development. *Gdf5* is not only an early marker for the interzone cells but is also functionally required for the formation of many synovial joints in the mouse limb (*7*). Deletion of both *Bmp2* and *Bmp4* in the embryonic limb mesenchyme caused joint fusion between the stylopod and the zeugopod, although molecular markers for joint development were not assessed (*8*). Reminiscent of the Bmp mutants, mice lacking Tak1, a mitogen-activated protein kinase kinase kinase (MAP3K) activated by Bmp signaling, also exhibited joint fusion at the elbow and knee, a defect linked with the loss of *Gdf5* at embryonic day 14.5 (E14.5), the earliest time point examined (*9*). Excessive Bmp signaling due to deletion of the Bmp antagonist Noggin also blocked early joint formation particularly in the digits, as evidenced by the lack of *Gdf5*-positive interzones, highlighting the sensitivity of joint development to Bmp dosage (*10*). Similarly, Tgfβ signaling is critical for digit joint development, as loss of the obligatory receptor Tgfβr2 in the limb mesenchyme resulted in lack of interphalangeal joints, due to the failure in maintaining *Gdf5* expression in the presumptive interzone beyond E14.5 (*11*, *12*). Thus, Bmp and Tgfβ are important signals in controlling interzone formation with apparent site specificities.

Other developmental signals and transcription factors have been implicated in the initiation of joint development. Ihh deletion led to unsegmented digits in the mouse embryo, due to an early arrest in interzone formation as *Gdf5*-positive cells failed to traverse the cartilage anlagen (*13*, *14*). Wnt/β-catenin signaling has been shown to suppress chondrogenic differentiation from the joint progenitors to ensure proper joint tissue formation (*15*). The zinc-finger transcription factors *Osr1* and *Osr2* exhibit overlapping expression with *Gdf5* at the onset of interzone formation, and deletion of both genes markedly reduces *Gdf5* in the presumptive interzone, resulting in lack of multiple joints at the elbow, the knee, the carpus and the tarsus (*16*). Most recently, Creb5 was shown to control *Gdf5* expression in the interzone cells and was indispensable for the formation of most synovial joints (*17*). Thus, multiple growth factors and transcription factors are critical in controlling interzone formation.

In contrast to such wealth of information above, little is known about the potential role of metabolic regulation in joint development. Earlier studies in mouse embryos showed that the hypoxia-inducible factor Hif1a is enriched in digit interzones of the digits reflecting normal local hypoxia, and when deleted, causes a delay in digit interzone formation (*18*, *19*). Joint fusion and cell death were also observed at the elbow of those mutant mouse embryos (*19*). However, a potential link between Hif1a and metabolic regulation in the interzone was not explored. Increasing evidence supports the importance of glucose metabolism in skeletal development and homeostasis (*20*). In particular, glucose uptake via Glut1 is critical for embryonic skeletal growth by ensuring proper proliferation and hypertrophy of the growth plate chondrocytes (*21*). In addition, impaired glucose uptake via Glut1 was linked with joint cartilage degradation in mouse models of osteoarthritis (OA), whereas forced expression of Glut1 ameliorated the damages to articular cartilage caused by OA (*22*, *23*). Tgfβ1, an important factor for maintaining joint cartilage homeostasis, has been shown to stimulate glycolysis in human articular chondrocytes (*24*). However, whether glucose metabolism also influences early joint development has not been investigated.

Here, we discovered that glycolysis is notably up-regulated in emerging interzone cells compared to the adjacent chondrocytes in knee joint primordia. Deletion of *Glut1* and *Glut3* with either *Prx1Cre* in preskeletal limb mesenchyme or *Col2Cre* in chondrocytes (including interzone precursors) blocked the genesis of interzone in mouse embryonic limbs. In contrast, *Glut1/3* deletion with *Gdf5Cre* in established interzone cells did not cause the same defect, indicating a transient requirement of heightened glycolysis before full establishment of interzone cells. Furthermore, blocking Tgfβ signaling by deletion of *Tgf*β*r2* with *Prx1Cre* partially phenocopied the loss of *Glut1/3* in causing interzone defects. Biochemically, Tgfβ stimulates *Glut1/3* expression and glycolysis in chondrocytes via activation of mTORC1 and Hif1a. Our data reveal that a transient increase in *Glut1/3* expression and glycolysis dependent on Tgfβ signaling is required for chondrocyte-to-interzone cell conversion during early limb joint development.

## RESULTS

### scRNA-seq identifies glycolytic feature in synovial joint interzone cells

**Fig. 1.**
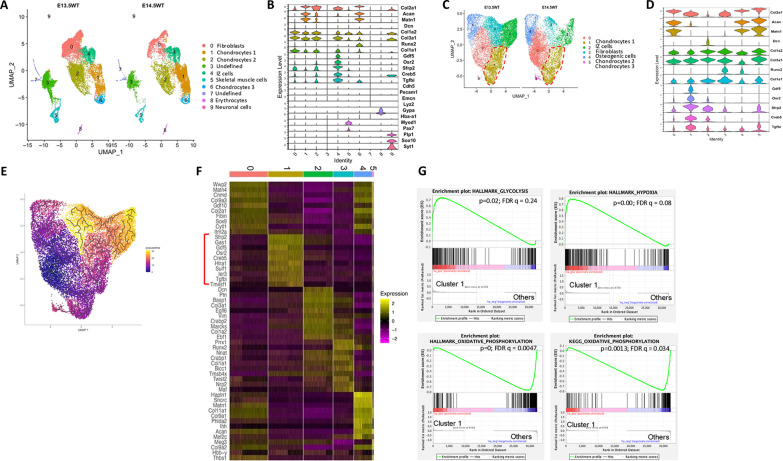
scRNA-seq identifies interzone cells in mouse embryonic knee joint primordia. (**A**) scRNA-seq uncovers diverse cell types in knee joint primordia of E13.5 versus E14.5 mouse embryos. IZ cells, interzone cells. (**B**) Violin plots of select marker genes for each cluster. (**C**) Reclustering of chondrocytes, fibroblasts, and interzone cells as identified in (A). The clusters are further analyzed in (D) to (H). (**D**) Violin plots of select marker genes for each cluster. (**E**) Monocle trajectory analysis with chondrocyte cluster 0 as origin. (**F**) Heatmap of top 10 DEGs enriched in each cluster. Red bracket denotes top genes in interzone cell cluster 1. (**G**) GSEA plots showing enrichment of glycolysis and hypoxia pathways but decrease in OXPHOS in interzone cells over all other clusters.

To gain a molecular signature of synovial joint interzone cells, we isolated the presumptive knee joint from E13.5 and E14.5 mouse embryos and performed single-cell RNA sequencing (scRNA-seq) (fig. S1A). Initial integrated analyses of data from the two time points identified a total of nine clusters, including minor populations of endothelial cells, erythrocytes, skeletal muscle cells, and neuronal cells (Fig. 1, A and B). Most of the cells were closely clustered together including three groups of chondrocytes (#1, 2, and 6) expressing high levels of *Col2a1*, *Acan* and *Matn1*, and fibroblasts (#0) enriched for *Col1a1*, *Col1a2*, and *Col3a1* (Fig. 1, A and B). Cluster 4, nestling between the fibroblasts and chondrocytes, uniquely expressed *Gdf5*, together with *Osr2*, *Sfrp2*, *Creb5*, and *Tgfbi*, and therefore represented interzone cells (Fig. 1, A and B) (*25*). To uncover additional heterogeneity, we reclustered the chondrocytes, fibroblasts, and interzone cells, and again yielded a single cluster of interzone cells (#1), three clusters (#0, 4, and 5) of chondrocytes, a fibroblast population (#2), but also a group of osteogenic cells (#3) expressing the most *Runx2* (Fig. 1, C and D). Trajectory analyses indicated that the interzone cells were more closely related to chondrocyte cluster 0 than the fibroblasts or osteogenic cells and could theoretically be derived from the chondrocytes (Fig. 1E). Analyses of differentially expressed genes (DEGs) showed that 261 genes were significantly enriched in the interzone cells compared to the other clusters as a whole (table S1). Among the top 10 most enriched are *Gas1*, *Htra1*, *Sulf1*, *Ier3*, and *Tm4sf1* that have not been previously studied in interzone cells (Fig. 1F). Also enriched were multiple genes in the core glycolysis pathway including *Ldha*, *Aldoa*, *Pkm*, *Eno1*, and *Gapdh* (table S1). Overall, scRNA-seq uncovered emergent interzone cells at different stages of development in the primordial knee joint of mouse embryos.

To gain further insights into the interzone cell cluster, we performed gene set enrichment analysis (GSEA) with the scRNA-seq data above. Compared with the other cell clusters, the interzone cells exhibited significant (*P* < 0.05 and *q* < 0.25) enrichment of seven HALLMARK pathways and suppression of five HALLAMRK pathways (fig. S1, B and C). Notably, hypoxia and glycolysis pathways were enriched, whereas oxidative phosphorylation (OXPHOS) was suppressed in the developing interzone cells (Fig. 1G). GSEA analyses also identified OXPHOS among the six Kyoto Encyclopedia of Genes and Genomes (KEGG) pathways significantly reduced in the interzone cells but no enrichment of KEGG pathways (fig. S1D). The results therefore identify increased glycolysis as a prominent feature of developing interzone cells compared to the neighboring cells including chondrocytes.

**Fig. 2.**
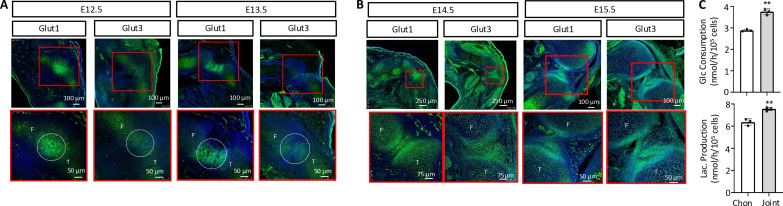
Interzone cells are enriched with Glut1 and Glut3 proteins and are highly glycolytic. (**A** and **B**) Immunofluorescence staining for Glut1 and Glut3 on frozen sections of hindlimbs. Dotted circles denote presumptive joint region. Boxed regions in lower magnification images were amplified at a higher magnification. (**C**) Glucose consumption and lactate production by knee joint primordial cells versus adjacent chondrocytes (Chon) from E13.5 hindlimbs. F, femur; T, tibia. **P < 0.01, *n* = 3, Student’s *t* test. Error bars: SD.

We next sought to verify the up-regulation of glycolysis in the developing interzone. Glut1 is the predominant glucose transporter in chondrocytes (*21*). Although scRNA-seq did not identify a significant increase of Glut1 mRNA in interzone cells over chondrocytes, immunostaining showed that Glut1 protein was up-regulated in the presumptive knee joint at both E12.5 and E13.5 (Fig. 2A). The increase of Glut1 was maintained in the developing articular cartilage and cruciate ligaments at E14.5 and E15.5 (Fig. 2B). Glut3, on the other hand, was expressed at similar levels in incipient interzone cells and chondrocytes at E12.5 and E13.5 but increased in articular cartilage and ligaments at E14.5 and E15.5 (Fig. 2, A and B). We next assessed glucose uptake ex vivo by incubating hindlimb explants isolated from E13.5 embryos without skin and soft tissues in a solution containing 2-[N-(7-nitrobenz-2-oxa-1,3-diazol-4-yl) amino]-2-deoxy-D-glucose (2-NBDG), a fluorescently labeled nonmetabolizable analog of glucose. The results showed a stronger signal in the presumptive joint region than the adjacent cartilage (fig. S1E). Last, to examine glycolysis directly, we isolated cells from the knee joint primordia and neighboring cartilage from E13.5 embryos. By using embryos with the genotype of *Gdf5Cre;Ai9*, we determined that approximately 40% of the isolated joint cells were tdTomato^+^ and therefore of the *Gdf5* lineage. During the brief culture period of in vitro testing, the joint cells consumed significantly more glucose and produced more lactate than the chondrocytes (Fig. 2C). Thus, both expression and functional studies support a more active glycolytic state in developing interzone cells than chondrocytes.

### Glut1 and Glut3 deletion disrupts interzone formation

**Fig. 3.**
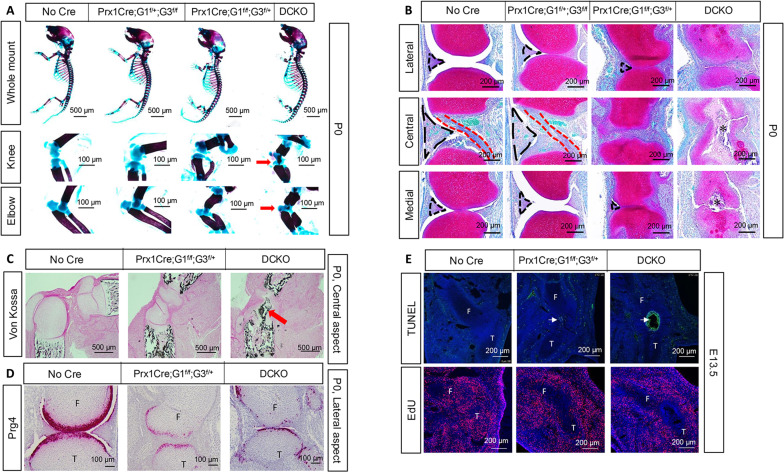
Deletion of *Glut1* and *Glut3* causes severe joint malformation. (**A**) Whole-mount skeletal staining at P0. Red arrows denote defects in knee and elbow joints. (**B**) Safranin O staining of sections through various planes of knee joints at P0. Black dashed line: meniscus; red dashed line: ligament. *: dead tissue. (**C**) Von Kossa staining of central sections through the knee joint. Red arrow: ectopic mineralization. (**D**) RNAscope of Prg4 on lateral sections of the knee. (**E**) EdU and TUNEL staining on knee sections at E13.5. White arrow denotes dead cells. G1, *Glut1*; G3, *Glut3*; DCKO, *Prx1Cre;Glut1*^*f/f*^*;Glut3*^*f/f*^. F, femur; T, tibia.

To determine the role of glycolysis in interzone formation, we deleted *Glut1* and *Glut3* in preskeletal limb mesenchyme with *Prx1Cre*, which becomes fully effective in the limb bud as early as E10.5, before the formation of mesenchymal condensations and cartilage anlagen (*26*). Whole-mount skeletal staining with alcian blue and alizarin red showed that newborn pups with one intact *Glut1* allele and no *Glut3* (*Prx1Cre;Glut1*^*f/+*^*;Glut3*^*f/f*^) were relatively normal, but those with one intact *Glut3* allele and no *Glut1* alleles (*Prx1Cre;Glut1*^*f/f*^*;Glut3*^*f/+*^) had notably shortened limb skeletal elements, especially the stylopods (femur or humerus) and zeugopods (tibia or radius/ulna) (Fig. 3A). The limbs were further reduced in *Prx1Cre;Glut1*^*f/f*^*;Glut3*^*f/f*^ double conditional knockout (DCKO) mice (Fig. 3A). Measurements of the femur confirmed that both the total length and the mineralized region were significantly reduced in both *Prx1Cre;Glut1*^*f/f*^*;Glut3*^*f/+*^ and DKO mice, but not in the *Prx1Cre;Glut1*^*f/+*^*;Glut3*^*f/f*^ littermates (fig. S2A). The findings not only confirm the essential role of *Glut1* in embryonic skeletal growth as previously reported but also uncover a role for *Glut3* when *Glut1* is absent (*21*).

Besides limb shortening, skeletal staining revealed defects at both knee and elbow joints in the mutant mice. Most notably, the DCKO mice failed to show clear separation between the stylopod and the zeugopod, but instead displayed ectopic mineralization in the presumptive joint region (Fig. 3A, red arrows). Staining of serial sections with Safranin O confirmed that the knee joint in the *Prx1Cre;Glut1*^*f/+*^*;Glut3*^*f/f*^ embryo was largely normal, but the one in *Prx1Cre;Glut1*^*f/f*^*;Glut3*^*f/+*^ mutants had only residual menisci and no cruciate ligament, with abnormally narrow separation between the femur and the tibia along the central plane of section (Fig. 3B). In the DCKO mice, no menisci were present and the cartilage elements remained connected at the central aspect of the prospective knee, with apparently dead tissue in the middle (Fig. 3B). Histological examination of the femur also revealed normal development in the *Prx1Cre;Glut1*^*f/+*^*;Glut3*^*f/f*^ newborn mice but profound shortening and disorganization of cartilage and bone in the *Prx1Cre;Glut1*^*f/f*^*;Glut3*^*f/+*^ or DCKO littermates (fig. S2B). Therefore, for subsequent analyses, we focused on the comparison between control and *Prx1Cre;Glut1*^*f/f*^*;Glut3*^*f/+*^ or DCKO mice. Von Kossa staining showed that the dead tissue in the knee of DCKO mice was mineralized, thus explaining the ectopic alizarin red staining observed earlier in the whole-mount skeletal preparation (Fig. 3C). Additional defects were observed in the articular cartilage of both *Prx1Cre;Glut1*^*f/f*^*;Glut3*^*f/+*^ and DCKO neonates, as RNAscope detected only weak signals of *Prg4* mRNA, in contrast to the robust expression in the normal articular chondrocytes (Fig. 3D). Severe joint defects were also observed at the elbow of *Prx1Cre;Glut1*^*f/f*^*;Glut3*^*f/+*^ and DCKO mutants even though segmentation of the skeletal elements appeared to have occurred (fig. S2C, upper panel). The phalange joints were virtually normal in either mutant, indicating site specificity of the dependence on *Glut1* and *Glut3* in synovial joint development (fig. S2C, lower panel). Overall, the data demonstrate a crucial role for glucose uptake in the development of knee and elbow joints.

We next determined the effect of Glut deletion on cell viability and proliferation. Terminal deoxynucleotidyl transferase–mediated deoxyuridine triphosphate nick end labeling (TUNEL) staining at E13.5 detected extensive cell death at the center of the presumptive knee joint in the DCKO mutant and also a few dead cells in the *Prx1Cre;Glut1*^*f/f*^*;Glut3*^*f/+*^ littermate but none in the wild-type embryo (Fig. 3E, upper panel). Similarly, cell death was notable in the knee at E16.5 or the elbow at E13.5 of the DCKO mutant (fig. S2D). On the other hand, EdU staining showed that cell proliferation in both the cartilage anlagen and the presumptive interzone was drastically reduced in the *Prx1Cre;Glut1*^*f/f*^*;Glut3*^*f/+*^ and DCKO mutants compared to the control (Fig. 3E, lower panel). A proliferation defect in growth plate chondrocytes was also observed at E16.6 in the two mutants but not in *Prx1Cre;Glut1*^*f/+*^*;Glut3*^*f/f*^ littermates (fig. S3, A and B). Thus, glucose uptake via Glut1 and Glut3 is essential for proliferation of both chondrocytes and prospective interzone cells but is uniquely required for survival of the latter.

### Glut1 and Glut3 deficiency impairs interzone genesis

**Fig. 4.**
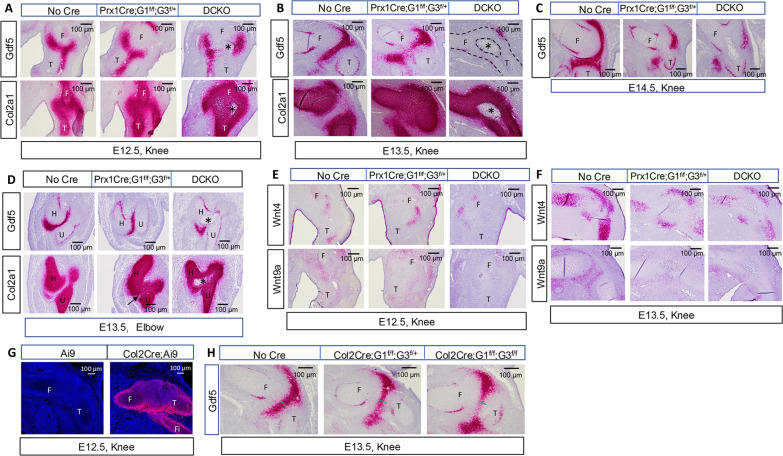
*Glut1/3* deletion disrupts interzone formation. (**A** to **D**) RNAscope for *Gdf5* and *Col2a1* on knee [(A) to (C)] or elbow (D) sections of *Prx1Cre*-mediated *Glut1/3* deletion mutants versus controls at E12.5 (A), E13.5 [(B) and (D)] and E14.5 (C). *: cell death area. (**E** and **F**) RNAscope for *Wnt4* and *Wnt9a* on knee sections at E12.5 (E) or E13.5 (F). (**G**) Confocal images of hindlimb sections showing red fluorescence in skeletal elements and knee joint primordia of *Col2-Cre;Ai9* but not *Ai9* embryos. (**H**) RNAscope of *Gdf5* on knee sections of *Col2Cre*-mediated *Glut1/3* deletion mutants versus controls at E13.5. Green line denotes normal height of developing interzone in the control embryo. G1, *Glut1*; G3, *Glut3;* DCKO, *Prx1Cre;Glut1*^*f/f*^*;Glut3*^*f/f*^. F, femur; T, tibia; H, humerus; U, ulna; Fi, fibula.

To gain molecular insights into the defects in joint formation caused by *Glut1/3* deletion, we performed comprehensive RNAscope analyses for *Gdf5* mRNA, a classic marker for both developing and established interzone cells, during the early stages of joint development. At E12.5, *Gdf5* mRNA demarcated the segmentation between the femur and tibia anlagen in controls and the level remained relatively normal in *Prx1Cre;Glut1*^*f/f*^*;Glut3*^*f/+*^ embryos (Fig. 3A). In the DCKO embryo, however, *Gdf5*-positive cells were largely absent along the prospective interzone area but were present in the immediately surrounding tissues, indicating a failure in interzone formation (Fig. 4A). On the other hand, *Col2a1* was abundant in most cells in the E12.5 DCKO presumptive joint region, except for a small cluster of likely dead cells at the center (Fig. 4A). Given their incipient nature at this stage, the newly emerging interzone cells in the control and *Prx1Cre;Glut1*^*f/f*^*;Glut3*^*f/+*^ embryos continued to express *Col2a1* (Fig. 4A). By E13.5, the interzone was further defined in the control, as indicated by the loss of *Col2a1* expression and increase in *Gdf5* expression (Fig. 4B). However, such changes did not occur in the *Prx1Cre;Glut1*^*f/f*^*;Glut3*^*f/+*^ embryos, which maintained strong *Col2a1* expression in the prospective interzone, whereas *Gdf5* mRNA was detected in fewer cells and more scattered than normal (Fig. 4B). The scattered pattern of *Gdf5*^*+*^ cells was also observed in E13.5 *Prx1Cre;Glut1*^*f/f*^ single CKO mutants even though the overall number of those cells appeared to be relatively normal, thus likely representing a less severe phenotype than that in *Prx1Cre;Glut1*^*f/f*^*;Glut3*^*f/+*^ embryos (fig. S3C). Most notably, *Gdf5* expression was virtually lost in the DCKO embryo, with only residual levels remaining at the periphery of the presumptive joint, whereas the center was occupied by dead cells (Fig. 4B). The defects in the *Prx1Cre;Glut1*^*f/f*^*;Glut3*^*f/+*^ embryo became more obvious at E14.5 when the center of the presumptive interzone failed to express *Gdf5* and showed signs of cell death reminiscent of, but less severe than, that in the DCKO mutant (Fig. 4C). Similarly, regarding the elbow joint of E13.5 embryos, the interzone in *Prx1Cre;Glut1*^*f/f*^*;Glut3*^*f/+*^ mutants was less developed than that in the control as indicated by the abnormal retention of *Col2a1* expression, whereas the prospective interzone in DCKO mutants was replaced by a zone of extensive cell death (Fig. 4D). The data therefore uncover an essential role for *Glut1/3* and glucose uptake at the early stages of interzone determination and formation.

Additional markers for early joint development were evaluated with RNAscope. *Wnt4* and *Wnt9a* expression progressively marked the peripheral joint tissues and the articular surface, respectively, in the control embryo from E12.5 to E13.5 (Fig. 4, E and F). Although *Wnt4* exhibited a relatively normal pattern in the *Prx1Cre;Glut1*^*f/f*^*;Glut3*^*f/+*^ or DCKO embryo, Wnt9a expression was not detected in the presumptive joint of either mutant at E12.5 or E13.5 (Fig. 4, E and F). Overall, the data indicate that disruption of glucose uptake via Glut1 and Glut3 in the precursors of interzone cells prevents interzone formation and subsequent joint tissue development.

As *Prx1Cre* targeted all limb mesenchymal cells, the joint defects above could potentially be due to non–cell-autonomous effects on the interzone. As the first and main cohort of interzone cells originated from chondrocytes, we tested the potential direct effect by deleting *Glut1/3* in chondrocytes with *Col2Cre*. Analysis with the *Ai9* reporter mouse confirmed that all presumptive joint cells at E12.5 were of the *Col2* lineage (Fig. 4G). Double deletion of *Glut1* and *Glut3* with *Col2Cre (Col2Cre;Glut1*^*f/f*^*;Glut3*^*f/f*^*)* markedly reduced the *Gdf5-*expressing emerging interzone cell population at the knee of E13.5 embryos, whereas partial deletion of *Glut1/3* (*Col2Cre;Glut1*^*f/f*^*;Glut3*^*f/+*^) resulted in a less severe but still noticeable reduction of the prospective interzone height (Fig. 4H). Consequently, by E18.5 the gene deletions led to a severe reduction of menisci in both mutants and to the absence of cruciate ligaments in the *Col2Cre;Glut1*^*f/f*^*;Glut3*^*f/f*^ but not *Col2Cre;Glut1*^*f/f*^*;Glut3*^*f/+*^ embryos (fig. S4). The results therefore support a direct role for the glucose transporters in interzone genesis and progression from chondrocytes.

**Fig. 5.**
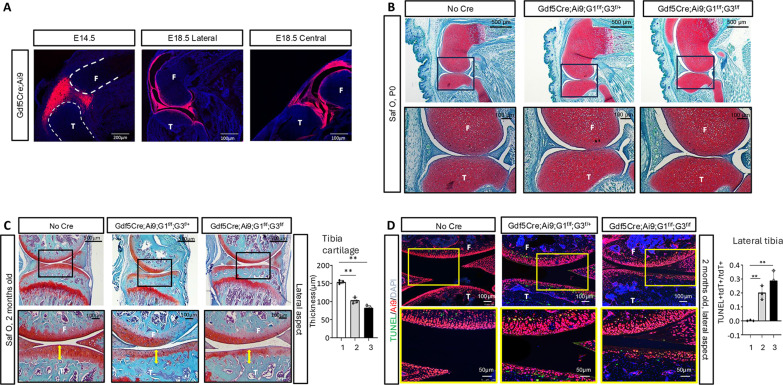
Deletion of *Glut1/3* with *Gdf5Cre* does not impede joint development but causes postnatal loss of articular cartilage. (**A**) Confocal fluorescent images of knee joint sections from *Gdf5Cre;Ai9* embryos. (**B** and **C**) Safranin O staining of knee sections from newborn (B) or 2-month-old mice (C) with genotypes as indicated. Boxed areas shown at higher magnification below. Double-headed arrows in (C) denote thinning of articular cartilage in tibial lateral condyle of mutant mice, with quantification shown to the right. (**D**) Confocal fluorescent images of knee joint sections from 2-month-old mice with genotypes as indicated. Red: *Gdf5-*lineage cells including articular chondrocytes; Green: TUNEL staining. Note increased dead articular chondrocytes (yellow nuclei) in the lateral tibia plateau of mutant mice, with quantification shown to the right. 1: No cre, 2: *Gdf5Cre; Ai9;G1*^*f/f*^*;G3*^*f/+*^, and 3: *Gdf5Cre;Ai9;G1*^*f/f*^*;G3*^*f/f*^. **P < 0.01, Student’s *t* test, *n* = 3 mice, one section from each mouse. Error bars: SD. F, femur; T, tibia.

We next sought to determine whether *Glut1* and *Glut3* continue to play a role after interzone cells are established. To this end, we chose to delete the transporters with *Gdf5Cre*, which, as previously reported, unlike the endogenous *Gdf5* expression in both developing and established interzone cells, elicits efficient recombination only from the three-layer interzone stage onward (e.g., E14.5 in the knee joint) (*27*). By crossing with the *Ai9* reporter mouse, we observed whole-body red fluorescence in a fraction of the progenies, indicating generalized Cre recombination in agreement with previous findings (*27*). In mice without the generalized Cre activity, *Gdf5Cre* was effective in targeting cells of the knee joint region at E14.5 and all knee joint structures at E18.5 (Fig. 5A). We therefore generated mice with *Gdf5Cre* deleting *Glut1* and *Glut3* while simultaneously activating *Ai9* expression and collected only mice without global red fluorescence for further analyses. In contrast to the deletions elicited by *Prx1Cre* or *Col2Cre*, deletion of both *Glut1* and *Glut3* with *Gdf5-Cre* did not overtly affect embryonic joint development, as indicated by the relative normalcy of knee joints at birth in *Gdf5Cre;Ai9;Glut1*^*f/f*^*;Glut3*^*f/+*^ or *Gdf5Cre;Ai9;Glut1*^*f/f*^*;Glut3*^*f/f*^ mice (Fig. 5B). However, at 2 months of age, articular cartilage of the lateral tibial condyle became notably thinner and flatter in both mutant mice than the control (Fig. 5C). The loss of articular cartilage was at least partly explainable by notable cell death among articular chondrocytes in both *Gdf5Cre;Ai9;Glut1*^*f/f*^*;Glut3*^*f/+*^ and *Gdf5Cre;Ai9;Glut1*^*f/f*^*;Glut3*^*f/f*^ mice, particularly clear in the tibial plateau as detected by TUNEL staining (Fig. 5D). It is currently unknown whether the cell death was secondary to the abnormal geometry of the tibial plateau that likely altered the distribution of mechanical force in the mutant knee. Furthermore, tissue damage to articular cartilage, reminiscent of that in OA, was observed in the medial tibial condyle of *Gdf5Cre;Ai9;Glut1*^*f/f*^*;Glut3*^*f/f*^ mice at 2 months of age (fig. S5). On the other hand, other joint tissues including cruciate ligaments and fat pads appeared to be largely normal in the mutants (fig. S5). Thus, *Glut1* and *Glut3* are dispensable for the survival and subsequent function of interzone cells once the cells become established but appeared to be necessary for the long-term maintenance of articular cartilage during postnatal life.

### Tgfβ signaling pathway is down-regulated in Glut1- and Glut3-deficient interzone cells

**Fig. 6.**
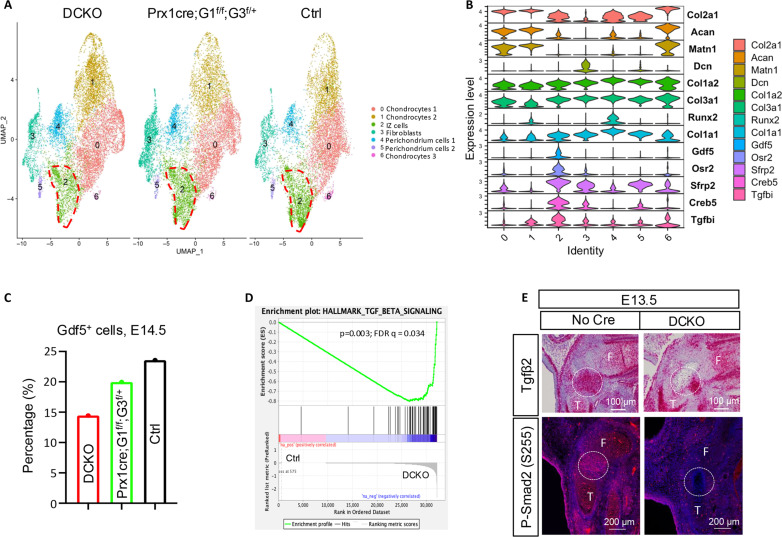
*Glut1/3* deletion suppresses Tgfβ signaling in *Gdf5*^*+*^ cells. (**A**) scRNA-seq of cells from joint regions of E14.5 embryos. DCKO, *Prx1Cre;Glut1*^*f/f*^*;Glut3*^*f/f*^. Red dashed lines denote *Gdf5*^*+*^ cells. (**B**) Violin plots of select marker genes for each cluster. (**C**) Relative abundance of interzone cells according to scRNA-seq. (**D**) GSEA reveals down-regulation of Tgfβ signaling pathway in *Gdf5*^*+*^ cells of DCKO versus Ctrl embryos. (**E**) RNAscope for *Tgf*β*2* (upper, dotted circles denote expression domain in joint region) and immunofluorescence for P-Smad2 (lower, dotted circles denote joint region) on knee sections at E13.5. Note the cell death in presumptive knee joint in DCKO samples. F, femur; T, tibia.

To gain mechanistic insights into the failure in interzone formation, we performed scRNA-seq with presumptive knee joint cells isolated from the *Prx1Cre;Glut1*^*f/f*^*;Glut3*^*f/+*^ or DCKO mutants versus control embryos (no Cre) at E14.5 (Fig. 6, A and B). Integrated analyses of the three datasets revealed that the percentage of Gdf5-expressing cells (cluster 2) was decreased from 23.5% in control (Ctrl) to 20% in *Prx1-Cre; Glut1*^*f/f*^*;Glut3*^*f/+*^ and 14.7% in DCKO (Fig. 6C). GSEA analysis revealed the Tgfβ pathway to be among the seven HALLMARK pathways being significantly down-regulated in the DCKO interzone cells compared to their normal counterparts (Fig. 6D and fig. S6A). scRNA-seq revealed that *Tgf*β*2* was not only significantly up-regulated in the developing interzone cells versus the other cells but also the highest expressed Tgfβ family gene there at E13.5 and E14.5 (table S1 and fig. S6B). Therefore, we examined *Tgf*β*2* expression in situ with RNAscope. Whereas *Tgf*β*2* was normally enriched in the primordial knee joint at E13.5, its expression was reduced to a small patch outside the dead zone in the DCKO sample (Fig. 6E). In addition, immunofluorescence staining showed that phosphorylation of Smad2, a readout for Tgfβ signaling and normally elevated in the knee joint over the neighboring cartilage at E13.5, was markedly reduced in both cartilage and the presumptive joint of DCKO embryos (Fig. 6E). Thus, the failure in interzone formation correlates with diminished Tgfβ signaling in presumptive interzone cells in vivo.

### Tgfβr2 deletion disrupts joint development

**Fig. 7.**
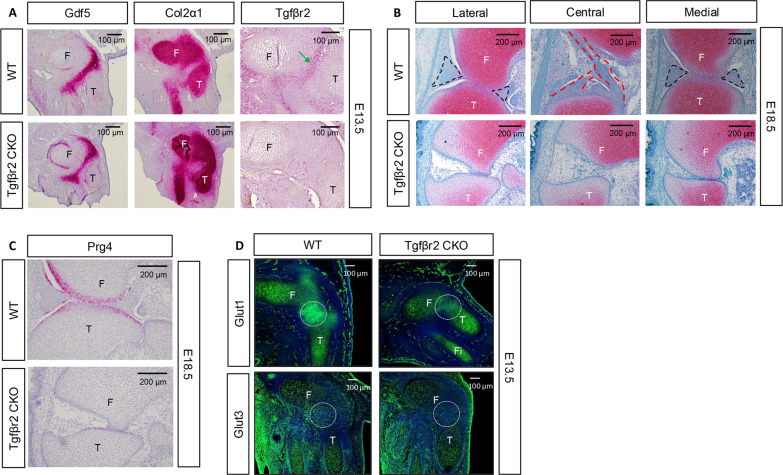
Deletion of Tgfβr2 disrupts interzone formation and knee joint development. (**A**) RNAscope on knee sections. Tgfβr2CKO: *Prx1Cre;Tgf*β*r2*^*f/f*^ at E13.5. Green arrow denotes enriched *Tgf*β*r2* expression in wild-type (WT) embryos but absent in Tgfβr2CKO. (**B**) Absence of meniscus (black dashed lines) and ligaments (red dashed lines) in the knee of Tgfβr2CKO at E18.5. (**C**) Loss of *Prg4* mRNA in the knee joint of Tgfβr2CKO at E18.5. (**D**) Immunofluorescence staining detects enhanced Glut1 and Glut3 expression in normal knee joint region (dotted circle) but not in equivalent areas of Tgfβr2CKO at E13.5. F, femur; T, tibia; Fi, fibula.

Impaired Tgfβ signaling upon *Glut1* and *Glut3* deletion prompted us to investigate the role of Tgfβ signaling in interzone formation. To this end, we deleted Tgfβr2, the obligatory type II receptor for all Tgfβ ligands, with *Prx1-Cre*. At E13.5, *Prx1Cre;Tgf*β*r2*^*f/f*^ mutants exhibited a narrower band of *Gdf5*-expressing interzone cells at the knee than normal (Fig. 7A). The reduced Gdf5 domain here was remarkably similar to that in the *Prx1-Cre;Glut1*^*f/f*^*;Glut3*^*f/+*^ (Fig. 4B) or the *Col2-Cre;Glut1*^*f/f*^*;Glut3*^*f/f*^ mutants (Fig. 4H). Also similar to the other mutants, the *Prx1Cre;Tgf*β*r2*^*f/f*^ embryos maintained a higher level of *Col2α1* expression in the presumptive interzone than normal (Fig. 7A). RNAscope also confirmed that *Tgf*β*r2* was normally enriched in the interzone but eliminated from the corresponding area in the *Prx1Cre;Tgf*β*r2*^*f/f*^ mutant (Fig. 7A). Moreover, histological sections at E18.5 revealed flattening of the articular surface along with absence of menisci and cruciate ligaments in the mutant knee (Fig. 7B). Confirming the defect in the articular cartilage, RNAscope showed that *Prg4* expression was virtually absent in the knee joint of *Prx1Cre;Tgf*β*r2*^*f/f*^ mouse (Fig. 7C). Thus, blocking Tgfβ signaling in the early limb mesenchymal progenitors, like deletion of *Glut1* and *Glut3*, impedes proper development of the interzone and its joint tissue derivatives.

As Ihh and Wnt signaling are implicated in early joint development, we next investigated their potential role in regulating interzone formation. The *Ihh*^*−/−*^ mice are known to lack joints in the digits and show abnormal morphology in wrist joints. However, RNAscope analyses of their knee joint showed robust *Gdf5* expression in the presumptive interzone at E13.5 (fig. S6C). Similarly, deletion of β-catenin with *Prx1Cre* did not preclude *Gdf5* expression in the developing knee joint despite the severe truncation of distal outgrowth (fig. S6D). Thus, compared to the other signals, Tgfβ plays a unique role in regulating the genesis of early interzone cells.

### Tgfβ2 stimulates glycolysis in chondrocytes

The similar defects observed in *Prx1Cre;Tgf*β*r2*^*f/f*^, *Prx1Cre;Glut1*^*f/f*^*;Glut3*^*f/+*^ and *Col2Cre; Glut1*^*f/f*^*;Glut3*^*f/f*^ mutants prompted us to examine whether Tgfβ signaling could regulate glucose uptake during interzone formation. Immunostaining at E13.5 revealed that Glut1, normally enriched in the emerging interzone compared to the neighboring cartilage, failed to up-regulate in the presumptive interzone of the *Prx1Cre;Tgf*β*r2*^*f/f*^ mutant (Fig. 7D). Likewise, Glut3 was diminished in the mutant joint primordia (Fig. 7D). Thus, loss of Tgfβ signaling results in the failure to up-regulate glucose transporters in presumptive interzone cells in vivo.

**Fig. 8.**
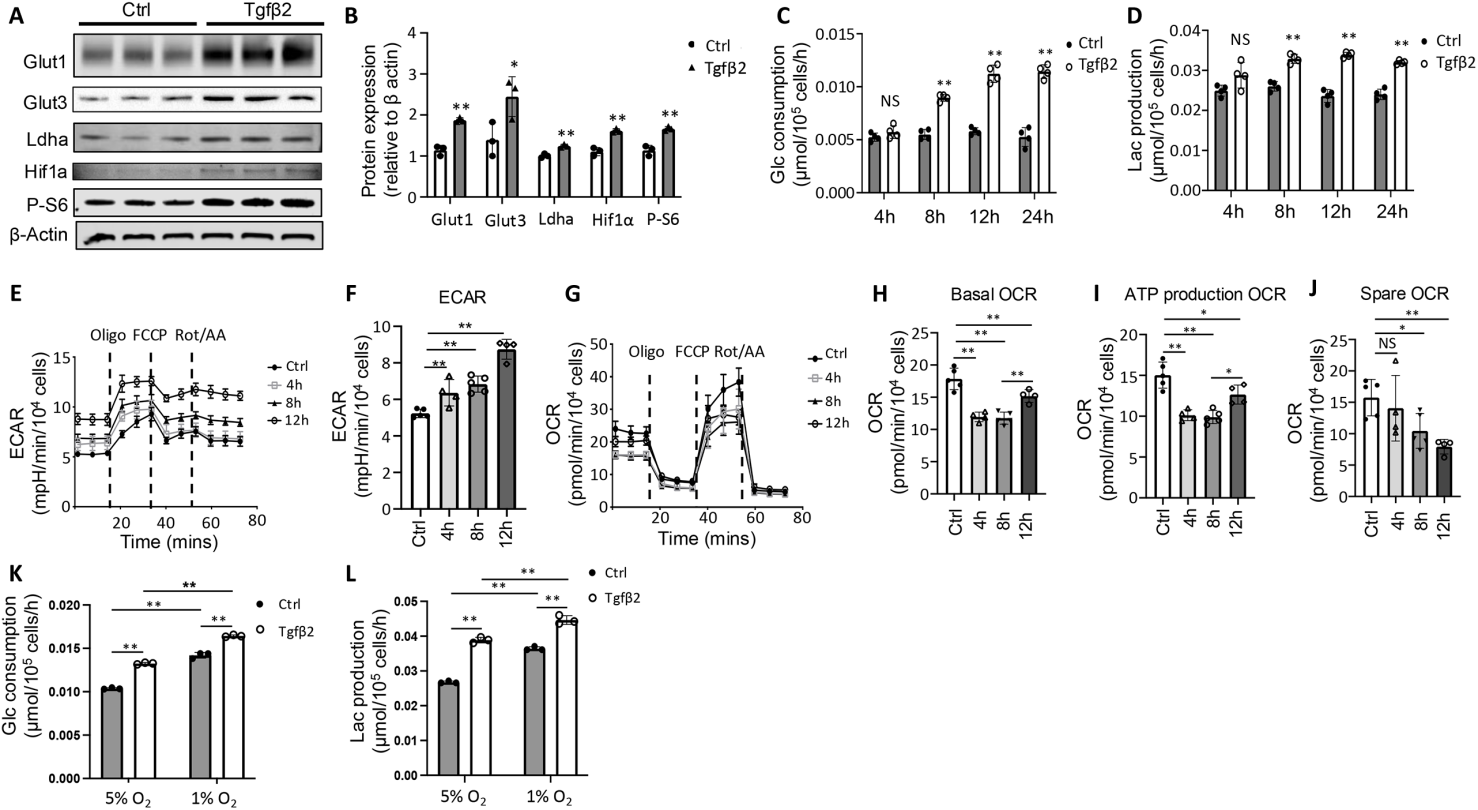
Tgfβ2 stimulates glycolysis in primary rib and limb chondrocytes. (**A** and **B**) Representative Western blot images (A) and quantification (B) from primary rib chondrocytes after 12 hours of Tgfβ2 or vehicle (ctrl) treatment. *N* = 3. (**C** and **D**) Glucose consumption (C) and lactate production (D) by primary rib chondrocytes following Tgfβ2 treatment for various durations as indicated. *N* = 4. (**E** to **J**) Seahorse assays in primary rib chondrocytes following Tgfβ2 treatment for various durations as indicated. *N* = 4 or 5. (**K** and **L**) Glucose consumption (K) and lactate production (L) by primary limb chondrocytes cultured in hypoxia (5 or 1% O_2_) and treated with Tgfβ2 or vehicle (Ctrl) for 12 hours. *N* = 3. *P < 0.05 and **P < 0.01, Student’s *t* test [(B) to (D)] or one-way ANOVA [(F) and (H) to (L)]. Error bars: SD.

As interzone cells originate from chondrocytes, we next tested whether Tgfβ might modulate glucose metabolism in chondrocytes themselves as a potential permissive cue for transition to interzone cells. Treatment of primary rib chondrocytes with recombinant Tgfβ2 for 12 hours markedly increased the protein levels of Glut1 and Glut3 (Fig. 8, A and B). Ldha, commonly associated with glycolysis, together with the master stimulator of glycolysis Hif1a, was also up-regulated by Tgfβ2 (Fig. 8, A and B). In addition, mTORC1 signaling, normally associated with increased protein synthesis, was stimulated by Tgfβ2, as indicated by increased phosphorylation of the ribosomal protein S6. Tgfβ2 stimulated glucose consumption and lactate production as early as 8 hours of incubation, even though the protein levels of Glut1 or Glut3 were not yet elevated at the early time point (Fig. 8, C and D and fig. S7A; we can leave this out if there are issues finding original blots). The increased glycolysis was confirmed by Seahorse assays, which showed significant increases in extracellular acidification rate beginning at 4 hours of Tgfβ2 treatment and continuing for at least 72 hours (Fig. 8, E and F, and fig. S7, B and C). On the contrary, both basal oxygen consumption rate (OCR) and the adenosine 5′-triphosphate (ATP) production OCR were significantly reduced after 4 hours of treatment, whereas the spare OCR was also reduced after 8 hours (Fig. 8, G to J). Both basal and ATP production OCR gradually recovered after 12 hours of Tgfβ2 treatment and returned to the pretreatment level after 72 hours, even though the spare OCR remained suppressed (fig. S7C). Thus, Tgfβ signaling stimulates glycolysis in chondrocytes, which may in turn support their transition to glycolysis-enriched interzone cells in response to other instructive signals.

As blocking Tgfβ signaling impaired interzone formation in the limbs, we next tested the potential metabolic effect of Tgfβ on embryonic limb chondrocytes. For this, we isolated chondrocytes from the developing tibias and femurs of E13.5 embryos. As nascent interzones have been shown to experience hypoxia, we cultured the cells with either 5 or 1% oxygen (*18*). As expected, reduced oxygen levels increased glucose consumption and lactate production by the cells with or without Tgfβ2 addition (Fig. 8, K and L). Tgfβ2 stimulated glycolysis in the cells cultured with either 5% or 1% oxygen (Fig. 8, K and L). The data therefore indicate that Tgfβ signaling can further promote glycolysis in the chondrocytes that prefigure the interzone cells and already experience hypoxia.

### Tgfβ activates glycolysis through mTOR and Hif1α

**Fig. 9.**
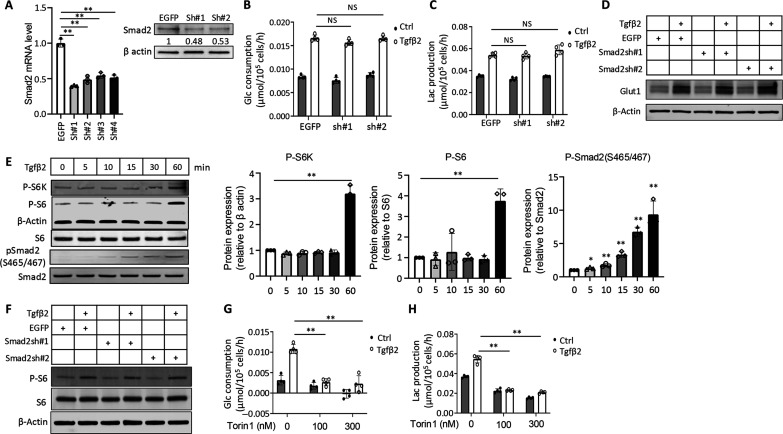
Tgfβ2 stimulates glycolysis through mTOR in primary rib chondrocytes. (**A**) Smad2 knockdown efficiency at mRNA (left) or protein level (right) by different shRNAs. Numbers in representative Western blot images indicate relative Smad2 levels normalized to β-actin. *N* = 3. (**B** and **C**) Quantification of glucose consumption and lactate production after 12 hour of Tgfβ2 or vehicle (Ctrl) treatment. *N* = 4. (**D**) Western blots showing effects of Smad2 knockdown on Glut1 after 12 hours of Tgfβ2 or vehicle (Ctrl) treatment. (**E**) Representative Western blot images and quantification of signal intensity showing temporal activation of Smad versus mTORC1 signaling by Tgfβ2. *N* = 3. (**F**) Representative Western blots images showing no effect by Smad2 knockdown on Tgfβ2-induced mTORC1 activation after 1 hour of Tgfβ2 or vehicle (ctrl) treatment. *N* = 3. (**G** and **H**) Effects of Torin1 on glucose consumption (G) and lactate production after 12 hours of Tgfβ2 or vehicle (Ctrl) treatment (H). *N* = 4. The inhibitor or its vehicle solution dimethyl sulfoxide (DMSO) was added to the cells for 12 hours (pretreatment) before either Tgfβ2 or its vehicle [0.1% bovine serum albumin (BSA)] was added. NS, not significant. *P < 0.05 and **P < 0.01, two-way ANOVA with Tukey’s multiple comparisons test. Error bars: SD.

We next investigated potential mechanisms mediating the glycolytic activation by Tgfβ. Canonical Tgfβ signaling is mediated by Smad2 or Smad3, but knockdown of Smad2 by >50% with two different shRNA constructs did not diminish the induction of glucose consumption or lactate production by Tgfβ2 in chondrocytes (Fig. 9, A to C). Similarly, Smad2 knockdown did not compromise the increase of Glut1 protein by Tgfβ2 (Fig. 9D). Attempts to knock down Smad3 with 10 different shRNA constructs failed to achieve adequate efficiency. Nonetheless, the data indicate that full activity of the canonical signaling pathway is not required for Tgfβ to activate glycolysis.

Tak1, a MAP3K activated by Tgfβ or Bmp signaling, was previously shown to control interzone development in the knee (*9*). That finding prompted us to examine the potential involvement of Tak1 in the metabolic regulation by Tgfβ. Chemical inhibition of Tak1 with 5Z-7-oxozeaenol, however, had no appreciable effect on the induction of glucose consumption by Tgfβ2, even though it modestly reduced both basal and Tgfβ2-induced lactate production (fig. S8, A and B). Similarly, the Tak1 inhibitor did not compromise the induction of *Glut1* mRNA or protein by Tgfβ2 even though it increased the basal Glut1 protein level independent of mRNA changes (fig. S8, C and D). On the other hand, Tak1 inhibition diminished Smad2 phosphorylation at both S255 and S465/467 normally induced by Tgfβ2 (fig. S8E). The results therefore rule out Tak1-MAPK activation as a major mechanism for the glycolytic stimulation by Tgfβ in chondrocytes.

**Fig. 10.**
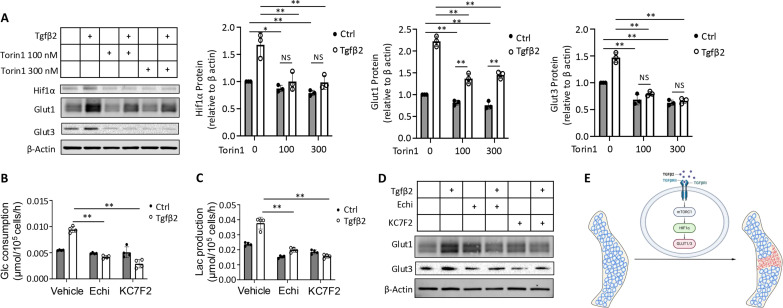
Tgfβ stimulates glycolysis via mTOR-Hif1a activation to facilitate transition of chondrocytes to interzone cells. (**A**) Representative images and quantification of Western blots showing effects of Torin1 on Hif1a and Glut proteins after 12 hours of Tgfβ2 or vehicle (Ctrl) treatment. *N* = 3. (**B** and **C**) Effects of Hif1α inhibitors echinomycin (Echi) or KC7F2 on glucose consumption (B) and lactate production (C) after 12 hours of Tgfβ2 or vehicle (Ctrl) treatment. *N* = 4. (**D**) Representative Western blot images showing effects of Hif1a inhibitors on Glut proteins after 12 hours of Tgfβ2 or vehicle (Ctrl) treatment. The inhibitors or their vehicle solution DMSO were added to the cells for 12 hours (pretreatment) before either Tgfβ2 or its vehicle (0.1% BSA) was added. NS, not significant. *P < 0.05 and **P < 0.01, two-way ANOVA with Tukey’s multiple comparisons test. Error bars: SD. (**E**) A model for Tgfβ signaling to facilitate interzone formation from chondrocytes via stimulation of glycolysis.

As mTOR-Hif1α activation has been shown to mediate glycolytic induction by Bmp in chondrocytes, we next examined its potential involvement in Tgfβ signaling (*21*). Western blot analyses showed that mTORC1 activation, as indicated by increased phosphorylation of S6K and S6, occurred after 1 hour of Tgfβ2 treatment, following the significant up-regulation of phospho-Smad2 detectable as early as 5 min after the treatment (Fig. 9E). However, knockdown of Smad2 had no effect on mTORC1 activation by Tgfβ2 (Fig. 9F). Inhibition of mTOR with Torin1 eliminated the induction of glucose consumption and lactate production by Tgfβ2, while also suppressing the basal rate of glycolysis (Fig. 9, G and H). In keeping with the reduced glycolysis, Torin1 notably blunted the induction of Glut1 protein and abolished that of Hif1α and Glut3 by Tgfβ2 (Fig. 10A). The changes in Glut1 or Glut3 protein levels were similar to those of Glut1 or Glut3 mRNA in response to either Tgfβ2 or Torin1, indicating that transcriptional regulation was likely the main mode of control for Glut1 and Glut3 expression in the current setting (fig. S8F). Furthermore, two different Hif1α inhibitors, echinomycin and KC7F2, completely eliminated the increase of glucose consumption and lactate production, as well as the induction of Glut1 and Glut3 proteins, by Tgfβ2 (Fig. 10, B to D). Likewise, the Hif1α inhibitors greatly suppressed *Glut1* mRNA levels regardless of Tgfβ2 treatment, demonstrating the critical dependence of *Glut1* transcription on Hif1α in chondrocytes (fig. S8G). Together, the study supports a model wherein locally enhanced Tgfβ signaling stimulates glycolysis via mTOR-Hif1a activation in preexisting chondrocytes to permit and sustain the transition to interzone cells in conjunction with other inductive signals (Fig. 10E).

## DISCUSSION

We have studied the role of glycolysis and Tgfβ signaling in synovial joint interzone formation. By using scRNA-seq, we discovered heightened glycolysis as a major metabolic feature of early developing interzone cells in the knee joint primordia of mouse embryos. Blocking glycolysis by deleting Glut1 and Glut3 in the early limb bud mesenchyme or incipient chondrocytes, both contributing interzone cell progenitors, greatly impaired the formation of a functional interzone. However, deletion of the same glucose transporters in established interzone cells with *Gdf5Cre* did not impede normal joint development, supporting a transitory requirement for increased glucose uptake during the genesis of the interzone from chondrocytes. The increase in glucose uptake is in part mediated by Tgfβ signaling, which appears to stimulate glycolysis via the mTOR-Hif1α axis in chondrocytes in vitro. This work therefore uncovers a hitherto unappreciated link between joint initiation and metabolic reprogramming during the transition from chondrocytes to interzone cells.

The in vitro data support the basic idea that Tgfβ markedly up-regulates *Glut1* mRNA via mTOR-Hif1a activation, but multiple effectors besides Glut1 likely mediate the activation of glycolysis by Tgfβ. This was evident as mTOR inhibition by Torin1 completely blocked Tgfβ2-induced glycolysis but only partially suppressed the induction of Glut1. Tgfβ1 was previously shown to stabilize Hif1a in cancer cell lines through down-regulation of PHD2 in a Smad-dependent manner, but here we show that in primary chondrocytes, knockdown of Smad2 had no effect on the Tgfβ2-induced glycolysis or Glut1 expression (*28*). Therefore, Tgfβ signaling may activate Hif1a signaling through different mechanisms depending on the cellular context. Moreover, the early induction of glycolysis following 8 hours of Tgfβ2 treatment occurred before Glut1 or Hif1a up-regulation at 12 hours after the treatment. Other mechanisms therefore must be responsible for the initial activation of glycolysis by Tgfβ and warrant further investigation. Last, future genetic studies are necessary to determine the role of Tgfβ-mTOR-Hif1a signaling in promoting glycolysis during interzone genesis in vivo.

In contrast to the knee and elbow joints, the interphalangeal joints appeared to be normal at birth in the absence of *Glut1* and *Glut3* expression. Thus, interzone formation in the digits seemed to be less dependent on glycolysis. Alternatively, other Glut family members may play a redundant role specifically in the digits to sustain sufficient glycolysis needed for interzone formation. Moreover, the interzone cells for the digit joints may exhibit greater metabolic plasticity by switching to other energy substrates when glucose uptake is limited. Previous studies support that the development of limb distal and proximal joints may obey different regulatory mechanisms. For example, loss of Gdf5 or Ihh mainly affects the distal joints (*7*, *13*, *14*). Thus, future studies are necessary to uncover potential differences in metabolic requirements between the interphalangeal and the more proximal joint formation in the limbs.

A requirement for Tgfβr2 in digit joint formation is well documented but the mechanism remains to be fully elucidated. One study found *Gdf5* expression to be up-regulated in developing digit interzone cells at E13.5 but suppressed at E16.5 when *Tgf*β*r2* was deleted, whereas another study reported normal levels of *Gdf5* at E13.5 and E14.5 but reduced expression at E15.5 in the mutant (*11*, *12*). Despite the differences, both studies support the view that Tgfβr2 is dispensable for the initial specification of *Gdf5*-expressing interzone cells in the digits but instead is required for their maintenance at later stages. Thus, the role of Tgfβr2 in interphalangeal joint development is likely distinct from that in the knee joint and may be separable from the regulation of glycolysis.

Deletion of *Tgf*β*r2*, unlike that of *Glut1* and *Glut3*, did not eliminate interzone formation at the knee. Thus, other signals in the presumptive joint sites may also play a role in up-regulating glycolysis to support interzone cell genesis. In this regard, Bmp, like Tgfβ, has been shown to stimulate glycolysis via activation of mTOR and Hif1a in chondrocytes (*21*). Further in support of potential involvement of Bmp signaling, deletion of both *Bmp2* and *Bmp4* in the limb bud mesenchyme caused joint fusion at the knee and elbow (*8*). In addition, the presumptive interzone has been shown to experience localized hypoxia resulting in up-regulation of Hif1a protein, which can in turn activate glycolysis. Of note, Tgfβ2 further activated glycolysis in embryonic limb chondrocytes cultured under hypoxic conditions in our experiment, and this is consistent with our finding that Tgfβ signaling and hypoxia activate Hif1a through different mechanisms. Overall, multiple growth factors together with the hypoxic environment are likely responsible for the heightened glycolytic state necessary for interzone genesis in the knee joint. Such increased energy production from glycolysis may be necessary for driving the morphological changes from chondrocytes to interzone mesenchymal cells. Alternatively, the metabolic reprograming could alter the epigenome via specific metabolites such as acetyl–coenzyme A or lactate to confer the gene expression signature of interzone cells (*29*, *30*). Future studies are warranted to explore those possibilities.

Last, it is important to consider the relative normalcy of knee joints in *Gdf5Cre;Glut1/3* mutants at birth in contrast to articular cartilage defects seen in the adults, including thinning of articular tissue and extensive cell death. Although we cannot rule out secondary effects from the altered joint geometry, the adult joint phenotype highlights the importance of the glucose metabolic regulators in long-term joint maintenance and function. Previous deletion of *Bmpr1a* with *Gdf5Cre* by others also led to postnatal degeneration of articular cartilage without impairing normal development of most joints (*27*). Likewise, in previous studies on primary cilia, we reported that the knee joints in *Gdf5Cre;Ift88*^*f/f*^ mouse mutants are largely normal at birth but acquire increasingly severe abnormalities over postnatal time, including reduced biomechanical modulus and abnormal zonal organization of articular cartilage (*31*). Last, the thinning of articular cartilage was also observed when we previously deleted *Glut1/3* in the superficial articular cartilage with *Prg4-CreER*^*T2*^ specifically in postnatal mice, supporting a direct need for the glucose transporters in postnatal articular chondrocytes (*23*). Together, the studies sustain the notion that multiple genes exert stage-specific functions in the postnatal life of articular cartilage. Future studies are necessary to determine whether Bmp signaling or primary cilia activity interacts with glucose metabolism in articular chondrocytes to maintain postnatal joint health.

## MATERIALS AND METHODS

### Mouse strains

All mouse work was approved by the Children’s Hospital of Philadelphia Animal Care and Use Committee (IACUC approval number IAC 21-001296). Mice were housed at 22°C with a 12-hour light cycle (6 a.m. to 6 p.m.) and free access to food and water. Both male and female mice were analyzed in the study. *Prx1Cre*, *Col2Cre* (line 3), *Gdf5Cre*, *Glut1*^*f/f*^, *Tgf*β*r2*^*f/f*^, *Ihh*^*−/−*^, and β*-catenin*^*f/f*^ mouse lines are as previously described (*13*, *26*, *27*, *32*–*35*). *Glut3*^*f/f*^ mice were derived from frozen sperms of *C57BL/6N-Slc2a3*^*tm1c(KOMP)Mbp*^ purchased from Mouse Biology Program at University of California, Davis. As *Prx1Cre* is known to recombine flox alleles in the female germline, all conditional knockout mice with *Prx1Cre* were generated by crossing double heterozygous males carrying *Prx1Cre* and one floxed allele of the target gene with homozygous floxed females. For each experiment, a minimum of three embryos for each genotype as indicated in the figures was analyzed after the genotypes were determined. Morphological or quantitative assessment of tissue sections was performed in a blinded fashion. Embryos with the same genotype were randomly selected with no exclusion and the representative findings are reported here.

### scRNA-seq and GSEA

For timed pregnancy, breeding pairs of mice were set up at 6 p.m. and separated at 12 a.m. immediately after plug checking. The time of plug detection was considered the beginning of embryonic development (E0). Cartilage anlagen were isolated from the hindlimbs of E13.5 or E14.5 embryos and cleaned of soft tissues by rolling on Kimwipe paper. Presumptive knee joint regions were excised under a dissecting microscope (Leica) and digested in collagenase D (4 mg/ml; Sigma-Aldrich, catalog no. 11088858001) on an orbital shaker at 120 rpm, 37°C for 20 min. Libraries were constructed with the Single Cell 3’ Reagent Kits version 3.1 (10x Genomics) and sequenced with a NovaSeq sequencer at the Center for Applied Genomics of The Children’s Hospital of Philadelphia. Cell Ranger was used for demultiplexing, extraction of cell barcode, and quantification of unique molecular identifiers (10x Genomics). Downstream analyses were performed with Seurat version 3 (*36*). In brief, cells with poor quality (total genes >6000 or <200, or mitochondrial genes >10%) were excluded. The highly expressed feature genes were determined using FindVariableFeatures() function before regressing out cell cycle genes. A linear transformation and subsequent principal components analysis were performed on the scaled data. Uniform manifold approximation and projection nonlinear dimensional reduction was used to visualize cell clusters. Wilcoxauc in Seurat pipeline was used to generate a preranked list either for the same cluster in two different experimental conditions (e.g., interzone cells in ctrl versus mutant embryos in Fig. 5D) or for one cluster against other clusters in the same setting (e.g., interzone cells versus all other clusters in Fig. 1C). The rank list was then used in GSEA with MsigDB gene sets (*37*).

### Primary chondrocyte cultures

Rib chondrocytes were isolated from neonatal mice at postnatal day 3 or 5. In brief, rib cages were dissected to be free of most surrounding tissues and then incubated with collagenase II (3 mg/ml; Sigma-Aldrich, P6885) in Dulbecco’s Modified Eagle Medium (DMEM; Life Technologies) for 20 min at 37°C on a shaker to remove the residual soft tissues. The ribs were then washed twice with phosphate-buffered saline (PBS) and further incubated in collagenase II (0.3 mg/ml) in DMEM overnight in an incubator at 37°C. The resultant chondrocytes were centrifuged and resuspended in custom-made MEMα freshly supplemented with 5.5 mM glucose, 2 mM glutamine, 1 mM pyruvate, and 10% fetal bovine serum (FBS; referred to as complete MEMα or cMEMα) (*38*, *39*). The cells were cultured in a regular incubator till ~90% confluence before being dissociated and reseeded at the desired density (see below) for each experiment. All experiments used passage one cells. Certain cell cultures were treated with recombinant Tgfβ2 (15 ng/ml; R&D Systems, 7346-B2) and/or chemical inhibitors including 100 or 300 nM Torin1 (Tocris Bioscience, 4247), 5 nM echinomycin (Sigma-Aldrich, SML0477), 10 μM KC7F2 (Sigma-Aldrich, SML1043), and 300 or 600 nM 5Z-7-oxozeaenol (MedChem Express, HY-12686).

Limb chondrocytes were isolated from the hindlimbs of E13.5 mouse embryos. Specifically, tibias and femurs were dissected and cleaned of soft tissues by rolling off a sheet of Kimwipe paper before being digested in collagenase D (4 mg/ml; Sigma-Aldrich, 11088858001) on an orbital shaker at 120 rpm, 37°C for 20 min. The cells were seeded with αMEM (Thermo Fisher Scientific, 41061037) containing 10% FBS in six-well plates at 3.5 × 10^4^/cm^2^ and cultured in a regular incubator. After 1 day, the cells were incubated overnight with fresh αMEM plus 10% FBS under 5% or 1% oxygen in a hypoxia incubator (BINDER, CB160). Subsequently, the media were replaced with fresh media with or without Tgfβ2 (15 ng/ml; R&D Systems, 7346-B2). The cells were further cultured with 5 or 1% oxygen for 12 hours before the media was collected for glucose and lactate measurements. The results were normalized to the final cell number counted at the conclusion of the experiment.

For shRNA knockdown experiments, lentiviral pLKO.1-based shRNA clones targeting *Smad2* or *Smad3* were obtained from High-throughput Screening Core at the University of Pennsylvania. Construct IDs were as follows: shEGFP, SHC005; Smad2 shRNA#1, TRCN0000089334; Smad2 shRNA#2, TRCN0000089335; Smad2 shRNA#3, TRCN0000089336; Smad2 shRNA#4, TRCN0000089337; Smad3 shRNA#1 TRCN0000089023; Smad3 shRNA#2 TRCN0000089024; Smad3 shRNA#3, TRCN0000089025; Smad3 shRNA#4, TRCN0000089026; and Smad3 shRNA#5, TRCN0000089027. The shRNA target sequence information can be accessed through the Broad Institute link (https://portals.broadinstitute.org/gpp/public/clone/search). The pLKO.1 shRNA constructs, together with packaging plasmids p△8.2 and pVSVG, were cotransfected into human embryonic kidney 293T cells by using Lipofectamine 3000 (L3000001, Thermo Fisher Scientific) for virus production. Primary chondrocytes were seeded in 24-well plates at 2.4 × 10^4^/cm^2^ and infected with lentiviruses at one transduction unit/cell for 16 hours. After culturing for 48 hours, the cells are used for total RNA extraction or metabolic assays.

### Metabolic assays

For Seahorse measurements, 60,000 rib chondrocytes were seeded with cMEMα as defined above in each well of 96-well Seahorse plates and cultured at 37°C with 5% CO_2_. After 6 hours, the culture media was replaced with fresh cMEMα supplemented with or without recombinant Tgfβ2 (15 ng/ml; 7346-B2, R&D Systems) and further cultured under the same conditions for various durations. Thereafter, the medium was replaced with the Seahorse assay medium (Agilent, 103575-100) containing 5.5 mM glucose, 2 mM glutamine, and 1 mM pyruvate, with (for cells with prior treatment of Tgfβ2) or without (for cells without the prior treatment) the addition of Tgfβ2; the plate was then incubated at 37°C without CO_2_ for 1 hour before Seahorse measurements. For the cells treated with Tgfβ2, the treatment time presented in figures included both the duration in cMEMα and that in Seahorse medium. Mitochondria stress tests were performed by sequential injections of 2 μM oligomycin, 3 μM carbonyl cyanide *p*-trifluoromethoxyphenylhydrazone, 1 μM rotenone, and 1 μM antimycin A.

For glucose and lactate measurements, rib chondrocytes were seeded with cMEMα in 24-well plates at 2.4 × 10^4^/cm^2^. After 6 hours, the medium was replaced with fresh cMEMα and the cells were cultured for varying durations of time before the medium was collected for measurements. Glucose and lactate concentrations were determined with a glucose (Sigma-Aldrich, GAHK20) or L-lactate assay kit (Eton Bioscience, 120001), respectively.

For glucose uptake assays ex vivo, the hindlimb skeleton was dissected form E13.5 embryos and cleaned of soft tissues with Kimwipe, before incubation in 100 μM 2-NBDG solution (PBS and 10% FBS) for 30 min (37°C, 5% CO_2_). The samples were then washed in PBS three times, 2 min each, followed by embedding in optimal cutting temperature and immediate sectioning at 15 μm in a cryostat machine (Leica). The images were captured with an SP7 confocal microscope (Leica).

For assaying glycolysis rates in joint primordia versus cartilage anlagen, the hindlimb skeletal elements of E13.5 embryos were dissected clean of soft tissues by rolling off a sheet of Kimwipe paper. The knee joint region based on morphology was severed from the rest of femoral and tibial cartilage with a scalpel under a dissecting microscope. Dissections from five to six embryos in the same litter were combined to collect sufficient joint versus cartilage tissues, which were separately digested in collagenase D (4 mg/ml; Sigma-Aldrich, catalog no. 11088858001) on an orbital shaker at 120 rpm, 37°C for 20 min. The cells were seeded with cMEMα in 24-well plates at 2.4 × 10^4^/cm^2^. After 6 hours, the media were replaced with fresh cMEMα and cultured for 12 hours before being collected for glucose and lactate measurements as described above. The results were normalized to the final cell number which was counted at the conclusion of the experiment.

### Whole-mount skeletal preparation

Whole-mount skeletal staining was performed as previously described (*21*). In brief, mouse embryos were skinned and eviscerated before fixation in 95% ethanol overnight and further incubation in acetone overnight. The embryos were then stained in solution containing ethanol, glacial acetic acid, Alcian Blue 8GX and Alizarin Red S for 3 days at room temperature on a slow rocker, and lastly cleared with 1% KOH mixed with an increasing amount of glycerol.

### Histology, TUNEL and EdU staining, and RNAscope

For routine histology, paraffin sections were prepared from embryonic limbs fixed overnight at room temperature in fresh 4% paraformaldehyde (PFA). For EdU labeling in vivo, pregnant mice were injected with EdU intraperitoneally at 10 μg/g body weight at 2 hours before euthanasia. To prepare paraffin sections for RNAscope, 4% PFA was made in DEPC-treated water to prevent RNAase activity. The fixed samples were then dehydrated by sequential incubation in 30% and 70% ethanol in PBS for 30 min each and lastly in 100% ethanol overnight. The dehydrated limbs were incubated in isopropanol twice and then in melted paraffin twice, for 30 min at each step. The processed limbs were embedded in paraffin and sectioned at 6 μm with a microtome (Leica).

For safranin O staining, deparaffinized sections were first stained in Hematoxylin QS (Sigma-Aldrich, 105174) followed by de-staining in acidic ethanol (1% HCl and 70% EtOH). Next, the sections were sequentially incubated in 0.1% Fast Green for 5 min, in 1% acetic acid for 10 s, and in 0.1% Safranin O for 5 mins. The sections were lastly rinsed in 95% ethanol twice before mounting with Acrymount Plus Mounting Media (StatLab) under a coverslip.

TUNEL and EdU staining was performed with DeadEnd Fluorometric TUNEL System (Promega, G3250) and Click-iT EdU Cell Proliferation Kit (Thermo Fisher Scientific, C10337), respectively, on deparaffinized sections according to manufacturer’s instructions. The sections were counterstained with 4′,6-diamidino-2-phenylindole (DAPI) before imaging with the SP7 confocal microscope (Leica).

RNAscope was performed on paraffin sections with RNAscope 2.5 HD Assay (Advanced Cell Diagnostics, catalog no.322350) by following the standard procedure. The sections were counterstained with hematoxylin and imaged under a light microscope (Nikon).

### Immunofluorescence staining

For immunofluorescence, frozen sections were prepared at 15 μm from embryonic limbs fixed in fresh 4% PFA for 2 hours at room temperature. The frozen sections were incubated in antibody diluent (Thermo Fisher Scientific, catalog no.003218) at room temperature for 1 hour before incubation overnight at 4°C with the primary antibodies diluted 1:200 in the antibody diluent. The primary antibodies were as follows: Glut1 (Ab115730), P-Smad2 (S255) (Ab188334) from Abcam, Glut3 (RRID: AB_2631293, a gift from K. Takata, Gunma University). After three washes with PBS, the sections were incubated for 1 hour at room temperature with the 1:500 diluted goat anti-rabbit immunoglobulin G (IgG) (H + L) cross-adsorbed secondary antibody (Invitrogen, A-11008 or A-21245). The sections were washed three times in PBS before staining with DAPI and mounting with ProLong Gold Antifade Mountant (Invitrogen, P36930). The images were captured with an SP7 confocal microscope (Leica).

### RT-qPCR

Total RNA extraction was performed using RNeasy Micro kit (Qiagen, 74004) according to the manual. One microgram of RNA was reverse transcribed to cDNA using the High-Capacity RNA-to-cDNA Kit (Applied Biosystems, 4387406). Subsequently, quantitative polymerase chain reaction (qPCR) was conducted using PowerUp SYBR Green Master Mix (Thermo Fisher Scientific, USA). β-Actin was used as internal control and 2^−ΔΔCT^ values were calculated for relative gene expression. Gene-specific primers are provided in table S1.

### Western blot analyses

Total protein was extracted using RIPA buffer containing 1/100 proteinase inhibitors and phosphatase inhibitors (Thermo Fisher Scientific, catalog no. 78442). Proteins were separated via electrophoresis in Nupage 4 to 12% bis-tris gel (Invitrogen, NP0322BOX) and transferred to the membrane (Sigma-Aldrich, IPFL00010) in the transfer buffer (Life Technologies, NP0006-1). The membranes were blocked with the Odyssey Blocking Buffer (PBS) (LI-COR Biosciences, USA, 927-70001) for 1 hour at room temperature. Subsequently, the membranes were incubated with primary antibodies overnight at 4°C. The following primary antibodies were used: Ldha (catalog no. 2012), S6 (catalog no. 2217), P-S6 (catalog no. 4858), P-S6K (catalog no. 9205), P-Smad2 (S465/467) (catalog no. 3108), Smad2 (catalog no. 5339), and β-actin (catalog no. 3700) from Cell Signaling Technology; P-Smad2 (S255) (Ab188334) and Glut1 (Ab1157730) from Abcam; Glut3 (20403-1-AP) from Proteintech; and Hif1α (NB100-479) from Novus. All primary antibodies were used at 1:1000 dilution except for Hif1α at 1:3000. After washing three times in tris-buffered saline with Tween 20, the membranes were incubated with the secondary antibody IRDye 800CW Donkey anti-Rabbit IgG (H + L) (catalog no. 926-32213) or IRDye 680RD Goat anti-Mouse IgG (H + L) (catalog no. 926-68070) from LI-COR Biosciences or horseradish peroxidase–conjugated anti-mouse IgG (catalog no. 7076) or anti-rabbit IgG (catalog no. 7074) from Cell Signaling Technology. Protein bands were visualized and quantified with the Odyssey DLx Imaging System or by chemiluminescence (Thermo Fisher Scientific, USA, catalog no. 34095) with the ChemiDoc MP imaging system (Biorad).

### Statistical analyses

Data were analyzed using two-tailed unpaired Student’s *t* tests or one-way analysis of variance (ANOVA) with Fisher’ least significant difference test or two-way ANOVA with Tukey’s multiple comparisons test, as indicated in figure legends.
