# Supplementary material for: Tgfβ signaling stimulates glycolysis to promote the genesis of synovial joint interzone in developing mouse embryonic limbs
Source: Sci Adv. 2025 Jan 8;11(2):eadq4991. doi: 10.1126/sciadv.adq4991 (PMC11708888; doi:10.1126/sciadv.adq4991)
Supplement: Supplementary file 1 — Figs. S1 to S8 Tables S1 and S2 [file sciadv.adq4991_sm.pdf]

Science Advances
AAAS

Supplementary Materials for

Tgfβ signaling stimulates glycolysis to promote the genesis of synovial joint interzone in developing mouse embryonic limbs

Chao Song *et al.*

Corresponding author: Fanxin Long, longf1@chop.edu

Sci. Adv. **11**, eadq4991 (2025)
DOI: 10.1126/sciadv.adq4991

This PDF file includes:

Figs. S1 to S8
Tables S1 and S2

Supplemental Figures

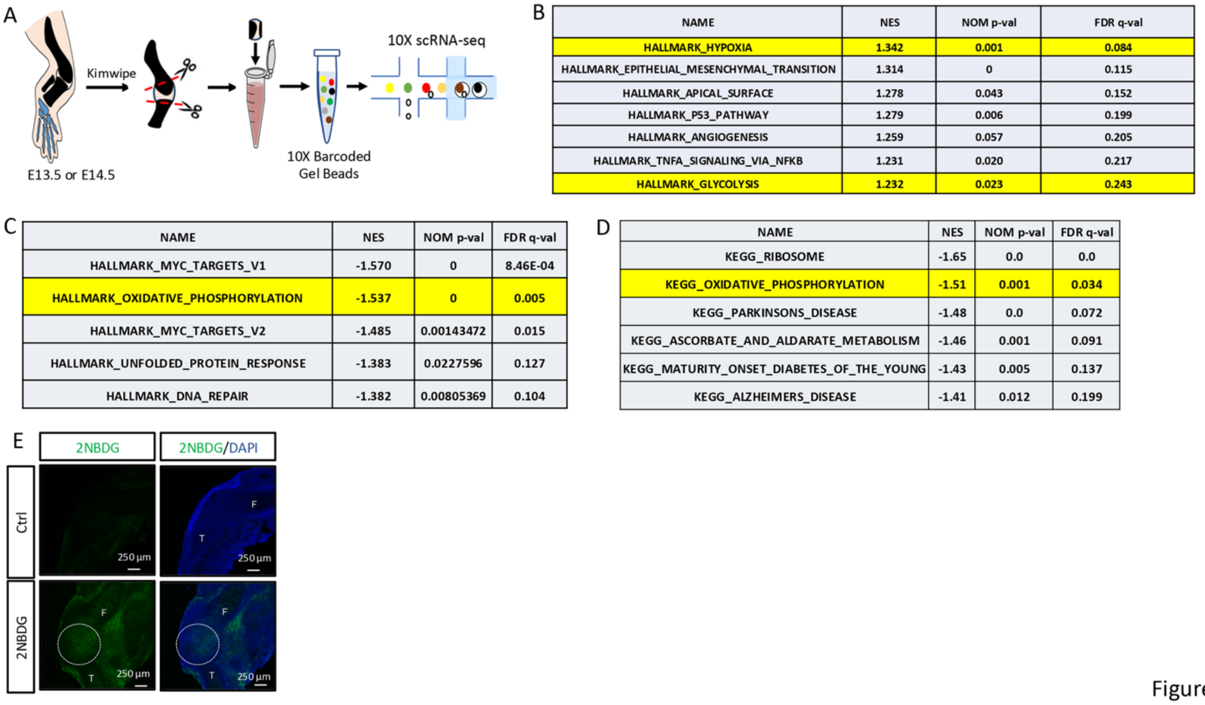

B

NAME	NES	NOM p-val	FDR q-val
HALLMARK_HYPOXIA	1.342	0.001	0.084
HALLMARK_EPITHELIAL_MESENCHYMAL_TRANSITION	1.314	0	0.115
HALLMARK_APICAL_SURFACE	1.278	0.043	0.152
HALLMARK_P53_PATHWAY	1.279	0.006	0.199
HALLMARK_ANGIOGENESIS	1.259	0.057	0.205
HALLMARK_TNFA_SIGNALING_VIA_NFKB	1.231	0.020	0.217
HALLMARK_GLYCOLYSIS	1.232	0.023	0.243

C

NAME	NES	NOM p-val	FDR q-val
HALLMARK_MYC_TARGETS_V1	-1.570	0	8.46E-04
HALLMARK_OXIDATIVE_PHOSPHORYLATION	-1.537	0	0.005
HALLMARK_MYC_TARGETS_V2	-1.485	0.00143472	0.015
HALLMARK_UNFOLDED_PROTEIN_RESPONSE	-1.383	0.0227596	0.127
HALLMARK_DNA_REPAIR	-1.382	0.00805369	0.104

D

NAME	NES	NOM p-val	FDR q-val
KEGG_RIBOSOME	-1.65	0.0	0.0
KEGG_OXIDATIVE_PHOSPHORYLATION	-1.51	0.001	0.034
KEGG_PARKINSONS_DISEASE	-1.48	0.0	0.072
KEGG_ASCORBATE_AND_ALDARATE_METABOLISM	-1.46	0.001	0.091
KEGG_MATURITY_ONSET_DIABETES_OF_THE_YOUNG	-1.43	0.005	0.137
KEGG_ALZHEIMERS_DISEASE	-1.41	0.012	0.199

Figure S1

Figure S1. Gdf5^{+} interzone cells are highly glycolytic.

(A) Experimental scheme of interzone isolation and scRNA-seq. (B) GSEA analysis showing HALLMARK pathways enriched in interzone cells over all other clusters. (C, D) GSEA analysis showing HALLMARK (C) and KEGG (D) pathways downregulated in interzone cells over all other clusters. Pathways related to metabolism are highlighted. (E) Images of 2-NBDG uptake in E13.5 hindlimb sections following incubation ex vivo. White dotted circles denote presumptive joint region. F: femur; T: tibia; M: skeletal muscle.

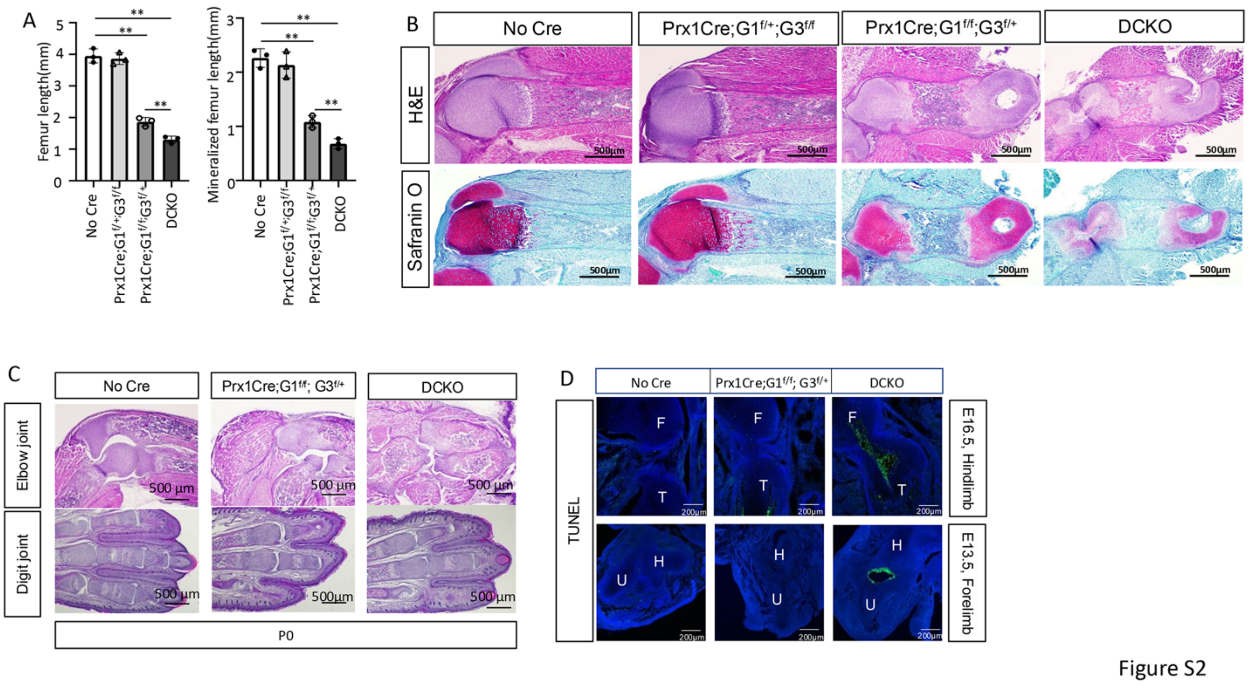

Figure S2. Glut1 and Glut3 deletion leads to severe joint malformation.

(A) Quantification of femur lengths measured from stained skeletons at P0. (B) H&E or Safranin O staining of sections through the femur and knee joint at P0. (C) H&E staining of forelimb sections showing malformed elbow joints but relatively normal digit joints at P0. (D) TUNEL staining of forelimb at E13.5 and hindlimb at E16.5. DCKO: Prx1Cre;Glut1$^{f/f}$;Glut3$^{f/f}$. F: femur; T: tibia; H: humerus; U: ulna. ** p<0.01, one-way ANOVA.

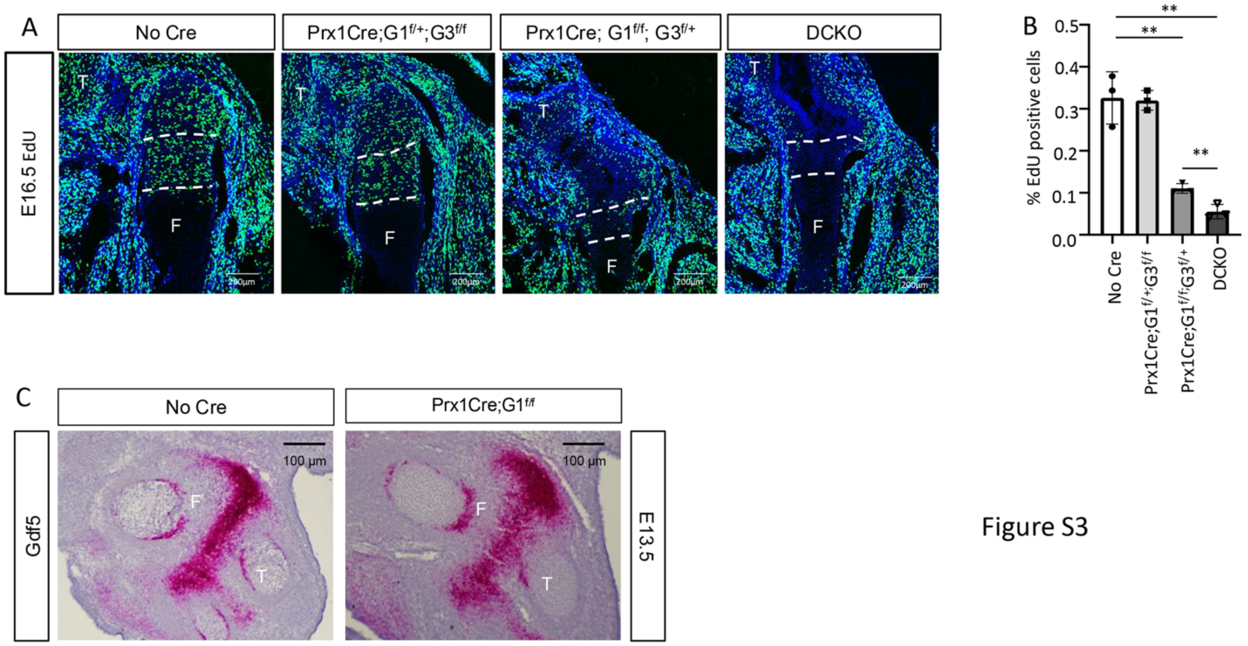

Figure S3

Figure S3. Deletion of Glut transporters impairs chondrocyte proliferation and interzone formation.

(A) Representative images of hind limb sections with EdU labeling in E16.5 embryos. Region of the femur between dotted lines was quantified in B. (B) Quantification of EdU labeling percentage among chondrocytes in marked region in A. N=3 embryos. One section per embryo was quantified. (C) RNAscope of Gdf5 on knee sections at E13.5. ** $p<0.01$, one-way ANOVA. Error bars: SD. F: femur; T: tibia.

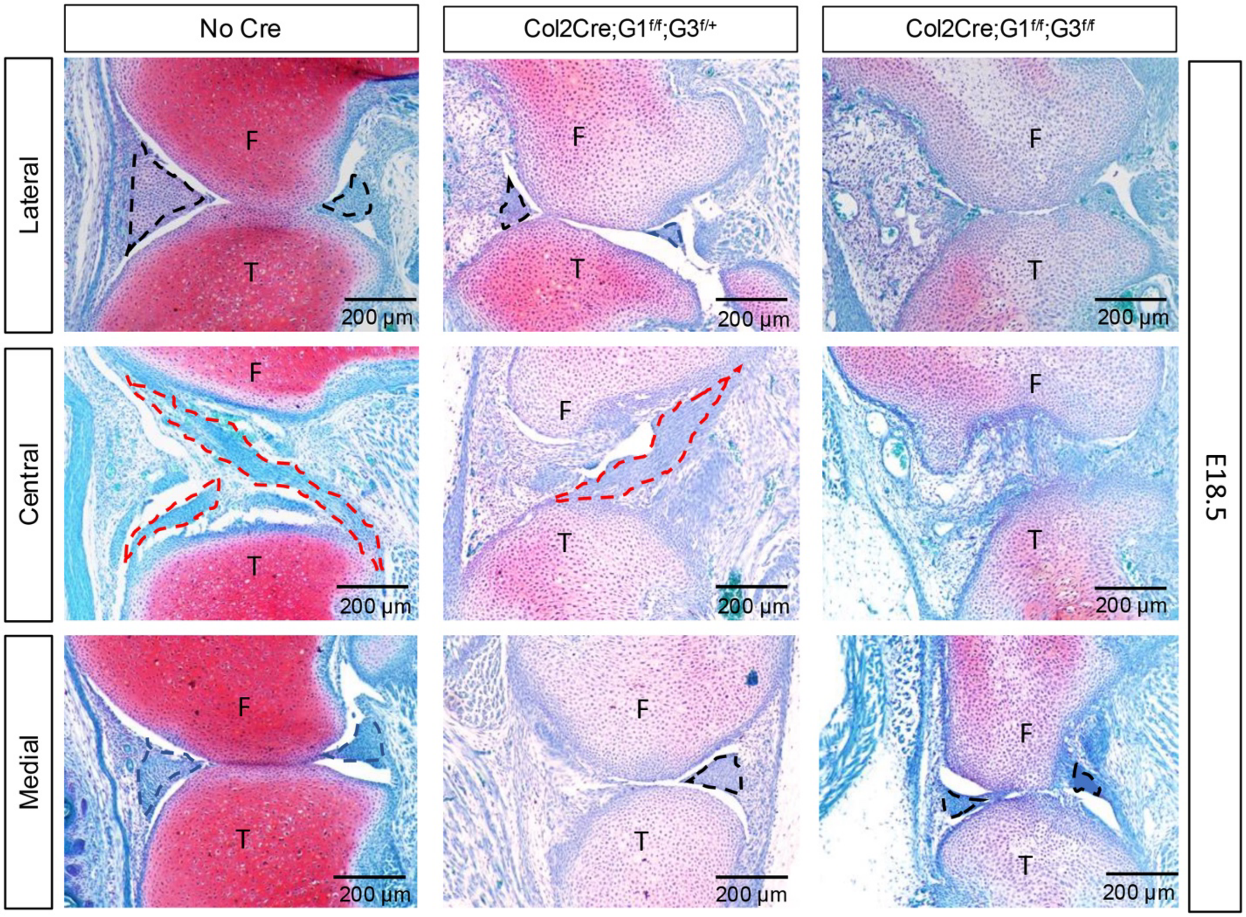

Figure S4. Deletion of Glut1 and Glut3 with Col2-Cre leads to severe joint malformation. Safranin O staining of sections through various planes of knee joints at E18.5. Red dotted lines denote cruciate ligaments. Black dotted lines denote menisci. F: femur; T: tibia.

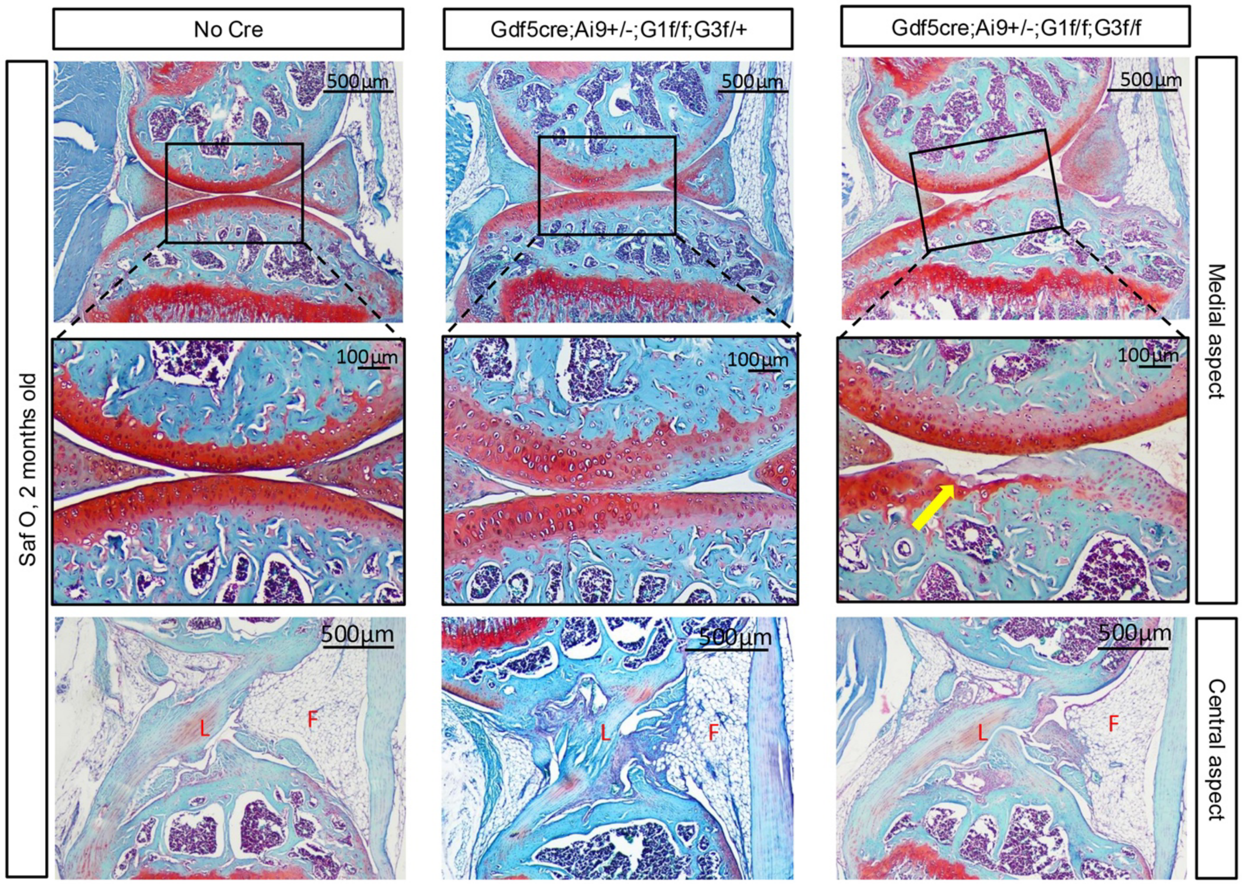

Figure S5. Deletion of Glut1 and Glut3 with Gdf5Cre leads to loss of articular cartilage in mice at 2 months of age. Sections through medial or central aspect of knee joint were stained with safranin O/methyl green. Boxed areas in medial section shown in higher magnification below. Arrow points to cartilage damage. L: ligaments; F: fat pads.

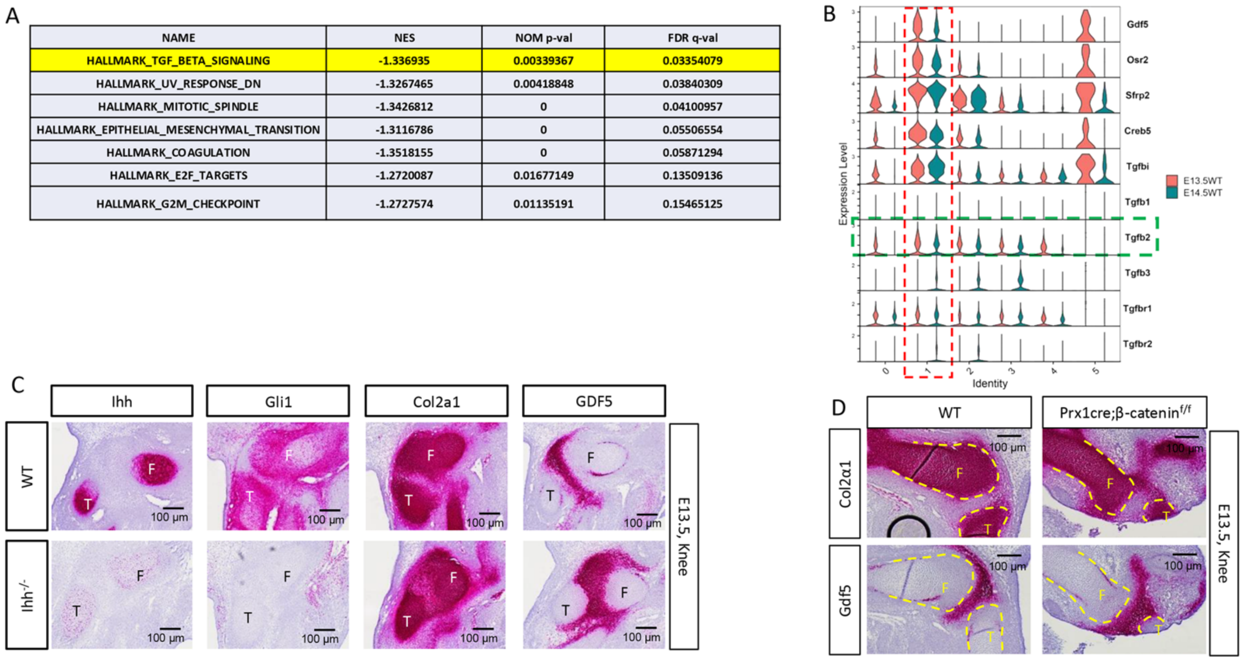

NAME	NES	NOM p-val	FDR q-val
HALLMARK_TGF_BETA_SIGNALING	-1.336935	0.00339367	0.03354079
HALLMARK_UV_RESPONSE_DN	-1.3267465	0.00418848	0.03840309
HALLMARK_MITOTIC_SPINDLE	-1.3426812	0	0.04100957
HALLMARK_EPITHELIAL_MESENCHYMAL_TRANSITION	-1.3116786	0	0.05506554
HALLMARK_COAGULATION	-1.3518155	0	0.05871294
HALLMARK_E2F_TARGETS	-1.2720087	0.01677149	0.13509136
HALLMARK_G2M_CHECKPOINT	-1.2727574	0.01135191	0.15465125

Figure S6. Tgfβ signaling regulates early joint development in the knee.

(A) GSEA reveals gene pathways impaired in $Gdf5^{+}$ cells of DCKO ($Prx1Cre;Glut1^{f/f};Glut3^{f/f}$) vs Ctrl based on scRNA-seq at E14.5. The Tgfβ pathway is highlighted. (B)Violin plots show expression of Tgfβ ligands and receptors among the different clusters identified by scRNA-seq in E13.5 and E14.5 wild-type joint primordia. (C, D) RNAscope detects no reduction of $Gdf5^{+}$ interzone cells in the knee of either $Ihh^{-/-}$ (C) or $Prx1Cre;β\text{-}catenin^{f/f}$ mutants at E13.5 (D). F: Femur; T:Tibia.

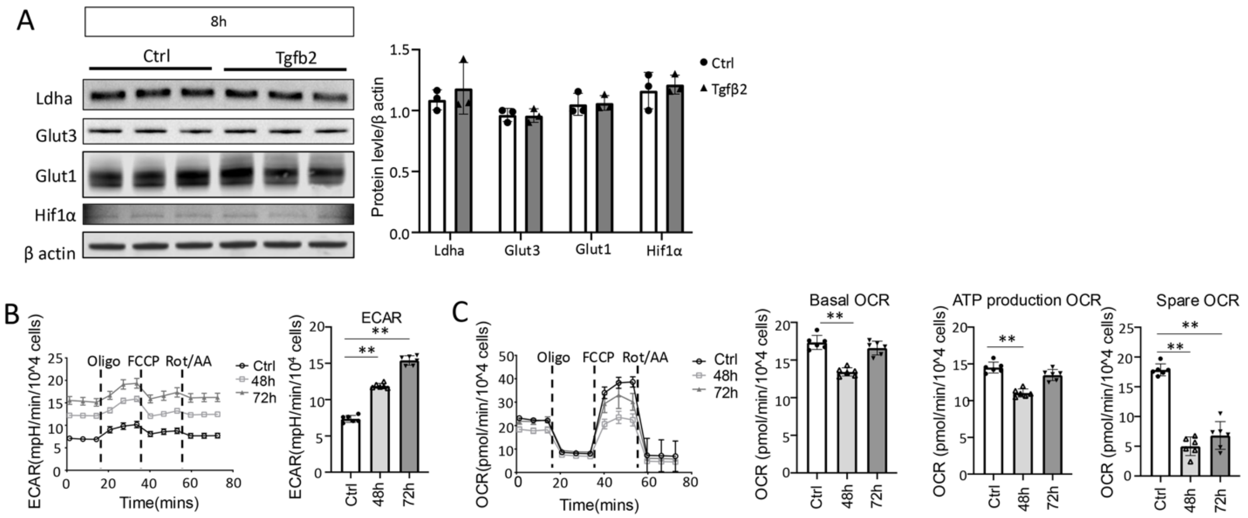

Figure S7. Tgfβ2 stimulates glycolysis in primary rib chondrocytes.

(A) Western blot images and quantification indicating no change in levels of glycolysis proteins in primary chondrocytes after 8 hrs of Tgfβ2 treatment. (B-C) Seahorse assays showing persistent stimulation of glycolysis by Tgfβ2 following prolonged incubation of up to 72 hrs. ** $p<0.01$, Student's t test, Error bars: SD.

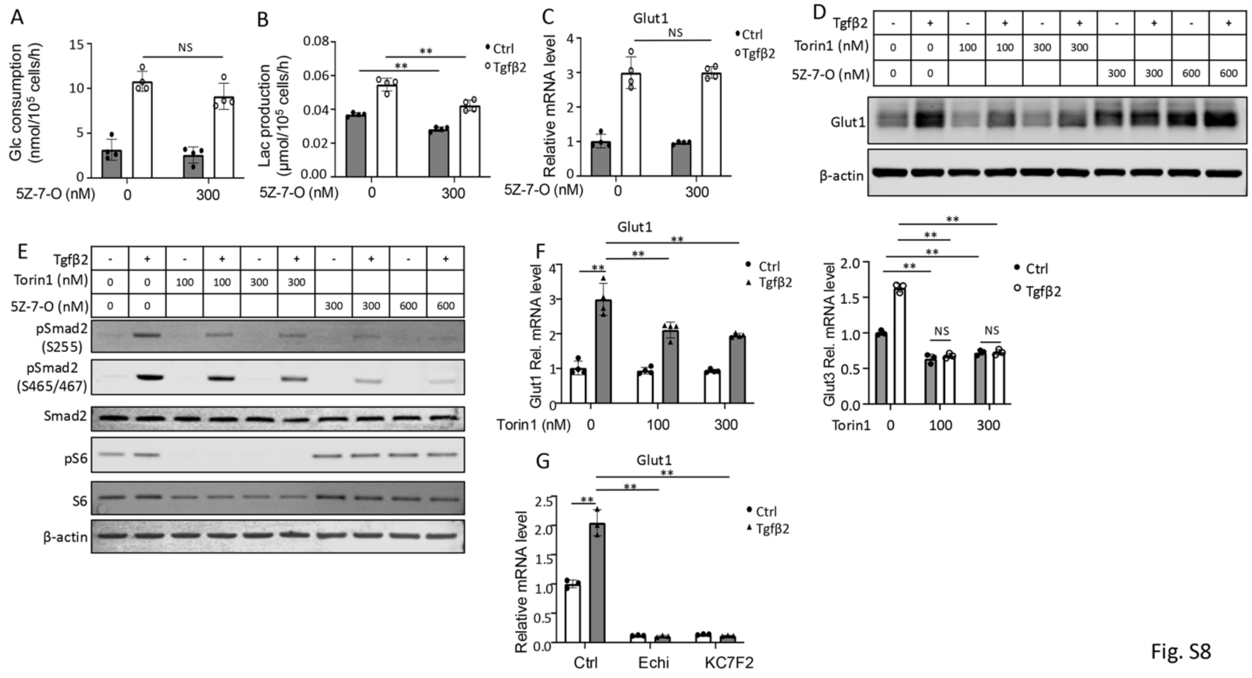

Fig. S8

Figure S8. Tgfβ2 stimulates glycolysis through mTOR/Hif1α in primary rib chondrocytes. (A, B) Little effect of TAK1 inhibitor 5Z-7-Oxozeaenol on induction of glucose consumption (A) or lactate production (B) by Tgfβ2 after 12 hrs. (C, D) Minimal effect of Tak1 inhibitor on Glut1 mRNA (C) or protein induction (D) by Tgfβ2 after 12 hrs. (E) Western blots showing effects of mTOR or Tak1 inhibitor on Smad and mTORC1 signaling with or without Tgfβ2 treatment for 1 hr. (F-G) RT-qPCR showing effects of mTOR inhibitor Torin1 (F) or Hif1α inhibitors echinomycin (Echi) and KC7F2 (G) on Glut1 or Glut3 mRNA levels with or without Tgfβ2 treatment for 12 hrs. For all inhibitor experiments, the cells were pretreated with either inhibitors or their corresponding vehicle (DMSO) for 12 hours before either Tgfβ2 or its vehicle (0.1% BSA) was added. ** $p<0.01$, two-way ANOVA, error bars: SD.

Supplemental Table I. RT-qPCR primer information

Gene	Forward	Reverse	Amplicon size (bp)
Slc2a1	GATTGGTTCCTTCTCTGTCGG	CCCAGGATCAGCATCTCAAAG	144
Slc2a3	CGCTTCTCATCTCCATTGTCC	TGAAGATAGTATTGACCACGCC	150
β actin	GTGACGTTGACATCCGTAAAGA	GCCGGACTCATCGTACTCC	245

Supplemental Table II. DEGs for interzone cells versus all other clusters in E13.5 knee joint

Positive log2FC indicates increased expression in interzone cells, p_val_adj <0.05, glycolysis genes in bold

	p_val	avg_log2FC	pct.1	pct.2	p_val_adj
Sfrp2	0	2.31067185	0.982	0.764	0
Gas1	0	2.0416307	0.966	0.707	0
Gdf5	0	1.97079182	0.804	0.434	0
Osr2	0	1.59660946	0.82	0.552	0
Creb5	0	1.4272022	0.881	0.599	0
Htra1	0	1.41344478	0.628	0.291	0
Sulf1	0	1.41159457	0.807	0.49	0
Ier3	0	1.4063391	0.688	0.36	0
Tm4sf1	6.64E-234	1.34790782	0.477	0.239	2.14E-229
Tgfbi	0	1.28239535	0.808	0.656	0
Sox4	0	1.20489855	0.935	0.809	0
Dusp1	4.10E-276	1.14039816	0.696	0.551	1.32E-271
Vcan	0	1.10509788	0.948	0.825	0
Tcf4	0	1.10458981	0.926	0.809	0
Jun	0	1.09500407	0.951	0.849	0
Lrrc17	1.27E-224	1.09174084	0.609	0.466	4.09E-220
Trps1	0	1.04631606	0.887	0.658	0
Foxp1	0	1.0101616	0.7	0.435	0
Spint2	0	0.98565306	0.735	0.427	0
Barx1	6.20E-195	0.96504929	0.651	0.47	2.00E-190
Khdrbs3	6.35E-242	0.96425165	0.637	0.507	2.05E-237
Rcan1	2.79E-256	0.94452967	0.526	0.292	9.00E-252
Zfp36l1	0	0.93163339	0.806	0.664	0
Pax9	8.95E-233	0.92268781	0.512	0.33	2.89E-228
Lrig3	0	0.88046598	0.875	0.701	0
Fbn2	0	0.86273732	0.913	0.846	0
Pdlim3	0	0.85971842	0.604	0.294	0
Cdh11	0	0.85380513	0.851	0.748	0
Tagln2	1.73E-186	0.82840387	0.716	0.639	5.58E-182
Robo1	0	0.82713169	0.678	0.482	0
Tsc22d1	0	0.82417233	0.973	0.932	0
Atf3	4.19E-224	0.81301896	0.497	0.272	1.35E-219
Scrg1	1.20E-162	0.79366322	0.482	0.25	3.88E-158
Epha7	4.27E-204	0.78732928	0.722	0.607	1.38E-199
Cdon	3.23E-259	0.78405989	0.568	0.34	1.04E-254
Meis2	1.53E-228	0.78137028	0.669	0.529	4.92E-224
Inhba	9.23E-130	0.78104647	0.459	0.31	2.98E-125
Man1a	4.04E-221	0.77098377	0.547	0.351	1.30E-216

Anxa2	1.27E-301	0.76294623	0.834	0.703	4.12E-297
Prss23	3.09E-63	0.75551628	0.499	0.423	9.99E-59
Sparc	0	0.74842687	0.964	0.905	0
Sema3c	2.27E-286	0.74438938	0.699	0.471	7.33E-282
Nfib	0	0.73488447	0.873	0.708	0
Fosb	9.38E-163	0.73224853	0.816	0.731	3.03E-158
Eif3h	0	0.67124869	0.98	0.967	0
Zfp503	2.59E-144	0.66687933	0.558	0.42	8.36E-140
Tnfaip8	5.15E-185	0.65474734	0.72	0.605	1.66E-180
Sulf2	1.14E-211	0.64789827	0.745	0.647	3.69E-207
C130073E24F	1.71E-76	0.63972831	0.425	0.299	5.52E-72
Arid5b	1.67E-228	0.63854052	0.721	0.572	5.40E-224
Pid1	4.94E-65	0.63202855	0.396	0.289	1.60E-60
Col12a1	7.49E-167	0.61543187	0.615	0.441	2.42E-162
Syt11	5.20E-177	0.61344465	0.548	0.397	1.68E-172
Efnb2	2.78E-114	0.61257349	0.544	0.429	8.96E-110
Tnfaip6	1.34E-106	0.60311515	0.599	0.49	4.34E-102
Runx1t1	9.01E-169	0.60183476	0.755	0.657	2.91E-164
Wnt4	7.45E-150	0.60035534	0.603	0.441	2.41E-145
Sgcd	1.42E-142	0.59477543	0.428	0.23	4.58E-138
Sap30	6.07E-192	0.58782861	0.633	0.46	1.96E-187
Smoc1	0	0.58480142	0.517	0.253	0
Ube2e3	1.44E-160	0.58111014	0.81	0.725	4.65E-156
Pamr1	2.97E-36	0.57213743	0.362	0.247	9.60E-32
Adgrl3	9.94E-202	0.57203682	0.763	0.629	3.21E-197
Fabp5	4.98E-73	0.56207669	0.792	0.768	1.61E-68
Tgfb2	2.82E-42	0.55543916	0.669	0.662	9.11E-38
Gas2	4.21E-124	0.55212482	0.767	0.727	1.36E-119
Lmo1	1.15E-158	0.55198697	0.446	0.265	3.72E-154
Aopep	1.18E-158	0.54149449	0.714	0.584	3.82E-154
Mab21l2	3.16E-164	0.53234718	0.805	0.681	1.02E-159
Fjx1	1.16E-61	0.53162942	0.499	0.414	3.73E-57
Ltbp3	3.10E-96	0.52970753	0.532	0.415	1.00E-91
Gli3	1.56E-121	0.52970744	0.621	0.497	5.03E-117
Pcdh10	1.04E-292	0.52794895	0.584	0.366	3.37E-288
Tmsb4x	4.79E-192	0.52279817	0.94	0.89	1.55E-187
Six1	1.01E-122	0.52180969	0.557	0.435	3.26E-118
Angptl1	5.25E-157	0.51508894	0.662	0.489	1.70E-152
Mif	2.09E-179	0.51244964	0.993	0.986	6.73E-175
Csrp2	2.69E-153	0.50488766	0.877	0.861	8.70E-149
Krt17	1.51E-15	0.50380773	0.247	0.128	4.88E-11

Lgals1	0	0.50354965	0.994	0.971	0
Fos	4.73E-90	0.50184407	0.912	0.905	1.53E-85
Cited2	7.42E-14	0.50172508	0.523	0.55	2.40E-09
Ctsl	7.26E-114	0.49880683	0.822	0.793	2.34E-109
Ackr3	1.83E-42	0.49832141	0.474	0.423	5.89E-38
Tle1	5.05E-137	0.49790621	0.669	0.547	1.63E-132
Jund	3.48E-187	0.48107857	0.996	0.989	1.12E-182
Ccdc80	6.93E-31	0.47266013	0.46	0.421	2.24E-26
Bnip3l	9.15E-93	0.47050922	0.841	0.792	2.96E-88
Bmp4	1.38E-52	0.46464254	0.492	0.429	4.45E-48
Tpm1	7.73E-120	0.46450595	0.822	0.791	2.50E-115
Adamtsl1	1.43E-28	0.46236194	0.557	0.54	4.61E-24
Zyx	2.21E-74	0.46030781	0.623	0.56	7.14E-70
Cd47	7.39E-123	0.4601161	0.484	0.319	2.39E-118
Igfbp5	1.05E-147	0.45850359	0.88	0.756	3.39E-143
Shisa2	2.66E-11	0.45775772	0.347	0.299	8.58E-07
Col5a1	3.24E-110	0.45593335	0.649	0.54	1.05E-105
Fzd8	6.58E-58	0.45328919	0.463	0.355	2.13E-53
Pdgfra	1.57E-61	0.44968875	0.717	0.718	5.07E-57
Tpi1	3.11E-115	0.4472011	0.945	0.925	1.00E-110
Nedd9	5.51E-140	0.44645535	0.43	0.253	1.78E-135
Pcdh18	6.87E-77	0.44596661	0.658	0.607	2.22E-72
Dleu2	1.10E-56	0.44567897	0.472	0.395	3.56E-52
Plxna4	6.89E-11	0.43864346	0.371	0.315	2.23E-06
S100a10	2.81E-137	0.43550648	0.727	0.596	9.06E-133
Fxyd6	1.38E-52	0.43417709	0.622	0.565	4.46E-48
Gem	2.71E-138	0.43022649	0.509	0.323	8.76E-134
Hmgb3	9.89E-131	0.42722579	0.889	0.848	3.19E-126
Epb41l3	2.17E-55	0.4234563	0.588	0.549	7.02E-51
**Ldha**	**1.29E-109**	**0.42049702**	**0.972**	**0.942**	**4.15E-105**
Rasl10a	3.94E-116	0.41775674	0.477	0.344	1.27E-111
Timp2	1.34E-96	0.41642595	0.673	0.587	4.33E-92
**Aldoa**	**7.38E-69**	**0.41267628**	**0.938**	**0.915**	**2.38E-64**
Arhgap31	5.57E-38	0.41041737	0.512	0.451	1.80E-33
Foxc1	6.82E-74	0.41032693	0.627	0.533	2.20E-69
Ift57	1.13E-154	0.40933596	0.574	0.402	3.65E-150
Mpped2	4.10E-21	0.40708693	0.783	0.792	1.32E-16
Egr1	9.16E-38	0.40606204	0.713	0.686	2.96E-33
Bex1	5.45E-114	0.40457792	0.64	0.517	1.76E-109
Pitx1	8.54E-118	0.40261876	0.914	0.896	2.76E-113
Tcp11l2	4.04E-91	0.40193579	0.49	0.386	1.30E-86

Igfbp2	7.33E-109	0.40029396	0.749	0.674	2.37E-104
Loxl2	1.98E-30	0.39622684	0.515	0.477	6.38E-26
Dkk3	3.06E-08	0.39508412	0.342	0.311	0.00098911
Tbx18	3.27E-98	0.39507367	0.625	0.545	1.06E-93
Dlc1	9.42E-38	0.39065331	0.558	0.516	3.04E-33
Rrad	5.54E-40	0.38854211	0.328	0.236	1.79E-35
Nfkbiz	1.03E-112	0.38174837	0.428	0.267	3.31E-108
Glcci1	1.05E-84	0.38157776	0.57	0.458	3.41E-80
Adamts1	1.62E-85	0.38053991	0.468	0.334	5.25E-81
Pkdcc	1.39E-46	0.37879906	0.765	0.744	4.50E-42
Fn1	1.23E-111	0.37862803	0.759	0.645	3.96E-107
Myoz1	3.45E-101	0.37713065	0.366	0.168	1.11E-96
Socs3	1.60E-44	0.37694806	0.674	0.618	5.18E-40
Nfil3	1.39E-50	0.37571682	0.523	0.449	4.50E-46
Plagl1	7.24E-74	0.37528358	0.818	0.709	2.34E-69
**Pkm**	**1.02E-67**	**0.37493166**	**0.955**	**0.947**	**3.31E-63**
Csrp1	1.41E-46	0.37364351	0.568	0.514	4.55E-42
Lrba	2.81E-54	0.37032188	0.496	0.419	9.08E-50
Ldlrad4	6.60E-22	0.37003713	0.442	0.394	2.13E-17
Xist	2.29E-90	0.3678406	0.876	0.831	7.38E-86
Ndufa4l2	3.51E-12	0.36467443	0.19	0.145	1.13E-07
Nxph1	2.08E-177	0.36457105	0.39	0.142	6.71E-173
Cpq	2.00E-31	0.36372053	0.429	0.344	6.45E-27
Myo1b	4.12E-07	0.36334424	0.519	0.552	0.0133144
Rgcc	1.19E-06	0.36149167	0.525	0.525	0.03856775
Rhoq	2.24E-19	0.36129089	0.442	0.4	7.22E-15
Rai14	6.92E-36	0.36129041	0.557	0.519	2.24E-31
Map1lc3a	6.03E-94	0.35813125	0.846	0.834	1.95E-89
Btg2	6.25E-73	0.35676949	0.613	0.504	2.02E-68
Chrdl1	8.14E-115	0.35471562	0.48	0.371	2.63E-110
Sox11	6.84E-57	0.350845	0.888	0.864	2.21E-52
Tle4	2.52E-21	0.3501784	0.581	0.561	8.13E-17
Rnd3	3.09E-60	0.34813209	0.632	0.58	9.97E-56
Slc38a1	5.38E-63	0.34545607	0.706	0.645	1.74E-58
Fstl1	1.07E-97	0.3442858	0.83	0.78	3.47E-93
Tsc22d2	6.84E-108	0.34179434	0.596	0.444	2.21E-103
Tafa2	2.14E-149	0.34122904	0.507	0.371	6.92E-145
Pmp22	3.43E-65	0.33794726	0.784	0.711	1.11E-60
Psd3	1.83E-51	0.3369518	0.39	0.283	5.90E-47
Svil	2.83E-34	0.33645301	0.634	0.619	9.13E-30
Dusp14	0	0.33553549	0.46	0.215	0

H1f0	2.49E-21	0.33489987	0.72	0.725	8.05E-17
Tnfrsf12a	9.86E-31	0.33052953	0.343	0.248	3.18E-26
Sema3a	5.42E-17	0.32871954	0.55	0.541	1.75E-12
Jmjd1c	1.98E-36	0.32597669	0.586	0.532	6.38E-32
**Eno1**	**4.08E-40**	**0.32583648**	**0.899**	**0.844**	**1.32E-35**
Lgr5	6.24E-51	0.32547254	0.313	0.167	2.01E-46
Gpc6	4.32E-52	0.32494487	0.686	0.586	1.39E-47
H3f3b	1.34E-237	0.32453574	1	1	4.33E-233
Nrep	1.33E-38	0.32328698	0.538	0.472	4.28E-34
Efna5	8.68E-65	0.3213701	0.503	0.426	2.80E-60
Pmepa1	4.34E-45	0.32101084	0.672	0.618	1.40E-40
Dab2	7.27E-24	0.32023061	0.548	0.516	2.35E-19
Ddah2	4.37E-77	0.31850127	0.856	0.862	1.41E-72
Olfml2b	1.31E-129	0.31845013	0.401	0.228	4.23E-125
Meg3	1.34E-88	0.31706474	0.981	0.955	4.34E-84
Dusp8	1.01E-17	0.31644776	0.362	0.314	3.27E-13
Nfic	2.21E-20	0.31616106	0.479	0.429	7.13E-16
Sox6	2.24E-47	0.3158704	0.718	0.613	7.24E-43
Sat1	1.03E-19	0.31551869	0.376	0.31	3.31E-15
Malat1	1.02E-133	0.31479892	0.999	0.999	3.28E-129
Lin7a	2.77E-89	0.31427561	0.465	0.355	8.93E-85
Pnrc1	4.95E-96	0.31083338	0.934	0.928	1.60E-91
Cadps2	6.21E-61	0.31038772	0.387	0.269	2.00E-56
Nfia	2.24E-41	0.3097313	0.56	0.491	7.24E-37
Tpd52	9.31E-17	0.30557947	0.447	0.425	3.01E-12
Hmcn1	7.62E-10	0.30529202	0.471	0.46	2.46E-05
Vps13b	1.44E-77	0.30443045	0.478	0.375	4.65E-73
Kras	7.61E-45	0.30264414	0.686	0.637	2.46E-40
Mfap2	2.19E-90	0.30238532	0.941	0.928	7.06E-86
Hoxd9	1.81E-11	0.30068068	0.535	0.507	5.83E-07
Tnni2	1.34E-13	0.29930242	0.18	0.181	4.33E-09
Bex4	3.03E-84	0.29858401	0.682	0.585	9.78E-80
Mat2b	7.70E-11	0.29818663	0.488	0.482	2.49E-06
Chst5	5.93E-21	0.29745243	0.252	0.279	1.91E-16
Rhob	7.51E-33	0.29466014	0.61	0.554	2.43E-28
Mpp7	3.45E-56	0.29465521	0.607	0.523	1.11E-51
Lhx9	6.99E-15	0.29399521	0.478	0.456	2.26E-10
Qk	1.25E-37	0.29197686	0.801	0.788	4.03E-33
Net1	9.20E-17	0.28875849	0.578	0.562	2.97E-12
Klf13	1.61E-17	0.28817295	0.465	0.43	5.20E-13
Fgfr2	7.67E-60	0.28771032	0.66	0.557	2.48E-55

Tax1bp3	1.80E-09	0.28707494	0.614	0.662	5.81E-05
Gm42418	2.20E-144	0.28610375	1	1	7.09E-140
Angptl2	2.50E-13	0.28382913	0.401	0.364	8.09E-09
Fhod3	3.69E-14	0.28149408	0.212	0.129	1.19E-09
Syne2	6.82E-70	0.28002565	0.604	0.538	2.20E-65
Dclk1	5.92E-13	0.27747017	0.379	0.33	1.91E-08
Tmx4	8.35E-32	0.27737951	0.545	0.484	2.70E-27
Smoc2	1.25E-28	0.27724643	0.549	0.474	4.03E-24
Disp1	1.68E-14	0.27691415	0.388	0.345	5.44E-10
Cdh13	1.68E-15	0.27554942	0.245	0.239	5.41E-11
Phactr2	7.90E-40	0.27528726	0.583	0.502	2.55E-35
Ltbp1	1.77E-08	0.27390373	0.469	0.464	0.00057003
Ccdc34	4.29E-27	0.27274313	0.777	0.777	1.38E-22
Ccnd1	2.23E-35	0.27226724	0.876	0.829	7.19E-31
Nbl1	2.74E-56	0.27208735	0.382	0.276	8.85E-52
Ly6h	1.44E-17	0.27087514	0.455	0.423	4.64E-13
Magi1	6.53E-115	0.26907326	0.517	0.438	2.11E-110
Espn	0	0.26714068	0.478	0.272	0
Six4	5.05E-43	0.26688809	0.438	0.363	1.63E-38
**Gapdh**	**1.08E-74**	**0.26549247**	**0.996**	**0.994**	**3.47E-70**
Khdrbs2	3.68E-32	0.26493601	0.41	0.346	1.19E-27
Ext1	4.00E-13	0.26378811	0.471	0.438	1.29E-08
Pcdh17	4.89E-13	0.26320339	0.376	0.332	1.58E-08
Ckb	5.18E-07	0.26288515	0.565	0.561	0.01673056
Tmem178	6.43E-46	0.26240219	0.338	0.22	2.07E-41
Sesn3	6.10E-17	0.26038394	0.58	0.57	1.97E-12
Stxbp6	5.71E-10	0.2601094	0.446	0.423	1.84E-05
Palld	7.15E-08	0.25971777	0.645	0.68	0.00230869
Dbn1	1.12E-41	0.25964704	0.772	0.753	3.61E-37
Tmed4	1.33E-33	0.25831642	0.556	0.494	4.30E-29
Prdx2	1.68E-118	0.25759544	0.997	0.994	5.42E-114
**Gpi1**	**1.14E-36**	**0.25722818**	**0.798**	**0.758**	**3.68E-32**
Cux1	6.41E-11	0.25629668	0.61	0.62	2.07E-06
Scx	5.39E-08	0.25628744	0.554	0.572	0.00174051
Eln	1.10E-18	0.2560702	0.394	0.324	3.56E-14
Mex3b	2.60E-16	0.25580597	0.618	0.617	8.40E-12
Bnip3	4.26E-13	0.25505412	0.59	0.549	1.37E-08
Nedd4l	6.45E-84	0.25440769	0.5	0.413	2.08E-79
Ctnnal1	2.14E-23	0.25406009	0.354	0.283	6.92E-19
Fam214a	1.14E-79	0.25357246	0.397	0.29	3.67E-75
Fam162a	3.84E-12	0.25203832	0.818	0.831	1.24E-07

1500004A13F	6.21E-214	0.25138054	0.482	0.331	2.00E-209
Trib1	5.44E-78	0.25126394	0.461	0.333	1.76E-73
Hoxd11	1.09E-07	0.25081083	0.472	0.53	0.00351687
Ism1	4.73E-115	0.25041481	0.486	0.354	1.53E-110
11-Sep	1.86E-66	-0.2504143	0.638	0.758	6.02E-62
1810058I24R	3.41E-64	-0.2508608	0.794	0.864	1.10E-59
Selenoh	2.22E-56	-0.2513756	0.828	0.894	7.16E-52
Panx3	1.64E-57	-0.2514611	0.092	0.266	5.29E-53
Fam204a	2.57E-106	-0.2526377	0.481	0.649	8.31E-102
P3h1	7.14E-61	-0.2529324	0.299	0.445	2.30E-56
Vcam1	3.04E-65	-0.2538738	0.464	0.548	9.81E-61
Edil3	6.28E-11	-0.253989	0.273	0.328	2.03E-06
Gnl3	6.32E-61	-0.2550491	0.577	0.687	2.04E-56
Crabp2	1.28E-26	-0.2551276	0.71	0.806	4.13E-22
Emp3	8.39E-44	-0.2553399	0.708	0.77	2.71E-39
Rab28	2.15E-111	-0.2557981	0.425	0.605	6.95E-107
Fzd9	2.23E-08	-0.2569342	0.309	0.329	0.00071848
Ebf3	1.51E-106	-0.2576421	0.36	0.525	4.88E-102
Tshz2	2.51E-24	-0.2582653	0.524	0.62	8.09E-20
Ppib	3.50E-114	-0.2583235	0.965	0.983	1.13E-109
Klhl13	2.12E-106	-0.2585518	0.314	0.459	6.84E-102
Chd3	5.91E-59	-0.2586077	0.742	0.852	1.91E-54
Hspg2	1.95E-56	-0.2586201	0.322	0.444	6.31E-52
Tpd52l1	5.56E-12	-0.2599973	0.53	0.536	1.79E-07
Rcn3	7.45E-77	-0.2601511	0.751	0.853	2.40E-72
P4ha1	1.56E-50	-0.2605534	0.546	0.632	5.04E-46
Ssr2	3.57E-69	-0.2605997	0.891	0.937	1.15E-64
Anxa5	1.49E-41	-0.261572	0.749	0.805	4.82E-37
Cspg4	5.59E-48	-0.2625052	0.389	0.481	1.80E-43
Eva1b	5.93E-93	-0.2638446	0.444	0.634	1.92E-88
Tmem263	1.46E-65	-0.2643843	0.583	0.693	4.73E-61
Dynll1	4.28E-107	-0.2656223	0.977	0.991	1.38E-102
Csgalnact1	5.79E-43	-0.2656485	0.247	0.36	1.87E-38
Tmem97	2.36E-59	-0.265732	0.447	0.553	7.62E-55
Pja1	1.11E-31	-0.2657972	0.453	0.535	3.57E-27
Tcf7l1	5.31E-72	-0.2660668	0.433	0.557	1.71E-67
Rplp1	4.55E-220	-0.2660729	0.996	0.996	1.47E-215
Tns3	2.04E-47	-0.2663493	0.423	0.516	6.59E-43
Ift20	4.27E-80	-0.2666353	0.699	0.816	1.38E-75
1500009L16R	3.92E-84	-0.2668856	0.417	0.555	1.26E-79
C1qbp	5.10E-72	-0.2669406	0.797	0.876	1.65E-67

Smad7	2.17E-54	-0.2682935	0.611	0.709	7.00E-50
Lpar4	1.66E-111	-0.2683595	0.4	0.583	5.37E-107
Fgfrl1	1.09E-33	-0.2683741	0.578	0.618	3.52E-29
Phgdh	1.97E-57	-0.2705265	0.788	0.844	6.37E-53
Tubb2a	3.37E-104	-0.2718899	0.336	0.519	1.09E-99
Kdelr3	3.68E-72	-0.2727507	0.396	0.538	1.19E-67
Fkbp9	6.18E-85	-0.2736733	0.434	0.598	2.00E-80
Raph1	7.44E-85	-0.2741965	0.292	0.436	2.40E-80
Eif4a1	9.72E-123	-0.2750517	0.951	0.985	3.14E-118
Hoxa11os	1.94E-191	-0.2762749	0.326	0.504	6.28E-187
Crip1	7.70E-61	-0.2764214	0.401	0.525	2.49E-56
Nin	1.14E-88	-0.2771574	0.249	0.394	3.68E-84
Cox4i2	2.20E-74	-0.278458	0.342	0.453	7.10E-70
Manf	5.99E-61	-0.2805904	0.832	0.894	1.93E-56
Tshz1	2.69E-42	-0.2817431	0.375	0.48	8.67E-38
Atp5e	1.06E-150	-0.2821562	0.977	0.991	3.42E-146
Papss1	5.10E-78	-0.283093	0.642	0.751	1.65E-73
Ctsz	2.17E-65	-0.2835287	0.57	0.685	7.02E-61
Ctnnbip1	2.65E-87	-0.2857184	0.694	0.821	8.55E-83
Oaf	1.44E-73	-0.2864655	0.659	0.757	4.64E-69
Serpine2	1.77E-18	-0.2867547	0.667	0.671	5.71E-14
Fibin	1.46E-36	-0.287004	0.718	0.738	4.71E-32
Etv5	6.46E-96	-0.2871097	0.163	0.338	2.09E-91
Aprt	1.09E-91	-0.2878785	0.813	0.904	3.50E-87
Snhg18	1.21E-96	-0.2882349	0.496	0.668	3.90E-92
Asap1	5.45E-66	-0.2891399	0.698	0.777	1.76E-61
Apbb2	3.87E-31	-0.2895278	0.345	0.421	1.25E-26
Clint1	2.30E-87	-0.2897604	0.586	0.728	7.41E-83
Auts2	3.72E-76	-0.2911404	0.873	0.933	1.20E-71
Gfpt1	1.03E-61	-0.2917576	0.519	0.623	3.34E-57
Batf3	1.23E-41	-0.2934329	0.483	0.556	3.97E-37
Btbd3	1.87E-45	-0.2945676	0.355	0.454	6.04E-41
Clcn5	1.40E-63	-0.2952936	0.341	0.473	4.53E-59
Wnt5a	3.66E-127	-0.2955818	0.457	0.609	1.18E-122
Gsn	1.17E-102	-0.2957789	0.353	0.503	3.78E-98
Ccnd2	1.51E-100	-0.2959859	0.718	0.883	4.87E-96
Atp5g1	6.13E-115	-0.2975071	0.891	0.954	1.98E-110
Kdelr2	1.75E-88	-0.2997433	0.728	0.831	5.65E-84
Matn3	7.01E-17	-0.3004633	0.209	0.289	2.26E-12
Klf2	4.20E-72	-0.3010666	0.647	0.752	1.36E-67
Slit3	3.96E-216	-0.3021727	0.382	0.551	1.28E-211

Egfl6	1.09E-126	-0.302786	0.234	0.395	3.53E-122
Rrbp1	1.35E-102	-0.3028358	0.903	0.954	4.37E-98
Tmem256	1.71E-109	-0.3045356	0.778	0.892	5.53E-105
Unc5c	5.32E-150	-0.3051892	0.338	0.522	1.72E-145
Lman1	2.50E-78	-0.3069813	0.697	0.796	8.08E-74
Mpp6	6.55E-114	-0.3088327	0.554	0.732	2.12E-109
Sec61b	3.04E-121	-0.3088659	0.961	0.981	9.80E-117
Ywhaq	4.44E-116	-0.3096201	0.923	0.964	1.43E-111
Vkorc1	3.96E-101	-0.3128071	0.532	0.698	1.28E-96
Pdlim4	3.27E-104	-0.3134572	0.569	0.706	1.06E-99
Trim47	1.37E-146	-0.3137606	0.153	0.345	4.41E-142
Ppa1	1.75E-54	-0.3142086	0.774	0.824	5.64E-50
Mme	0	-0.3150517	0.218	0.499	0
Limd2	2.05E-104	-0.3154082	0.686	0.799	6.63E-100
Lrrc59	2.55E-83	-0.3161314	0.667	0.764	8.23E-79
Peg3	4.16E-68	-0.3162803	0.932	0.961	1.34E-63
Ostc	8.97E-134	-0.3218625	0.901	0.956	2.89E-129
Reep5	5.99E-96	-0.3227383	0.618	0.76	1.93E-91
Igf2r	8.30E-96	-0.3248359	0.501	0.669	2.68E-91
Anxa1	5.42E-89	-0.3260736	0.062	0.246	1.75E-84
Bicc1	1.15E-92	-0.3262659	0.339	0.505	3.71E-88
Col23a1	5.47E-97	-0.3291914	0.45	0.576	1.77E-92
BC006965	2.94E-09	-0.3292151	0.318	0.343	9.50E-05
Hspd1	1.19E-105	-0.3304478	0.847	0.925	3.84E-101
Fam104a	2.73E-106	-0.3338547	0.715	0.821	8.83E-102
Ntm	6.62E-26	-0.3356166	0.191	0.305	2.14E-21
Lef1	3.56E-33	-0.335712	0.249	0.331	1.15E-28
Sorbs1	5.15E-76	-0.3379628	0.236	0.367	1.66E-71
Klf5	6.18E-64	-0.3392414	0.27	0.387	2.00E-59
Ost4	3.35E-109	-0.3393292	0.768	0.869	1.08E-104
Prrx2	6.85E-241	-0.3397992	0.43	0.623	2.21E-236
Bcl2	1.12E-133	-0.3424244	0.544	0.678	3.63E-129
Inka1	2.61E-88	-0.3441868	0.451	0.577	8.42E-84
Msi2	3.08E-92	-0.3443778	0.406	0.545	9.96E-88
Gadd45g	2.79E-47	-0.3457912	0.434	0.517	9.02E-43
Col27a1	2.32E-31	-0.3528747	0.638	0.638	7.48E-27
Ctdspl	1.87E-65	-0.3612773	0.58	0.642	6.04E-61
Rap2c	3.68E-119	-0.3628551	0.49	0.644	1.19E-114
Sh3bgrl3	1.42E-127	-0.3638534	0.808	0.898	4.59E-123
Ebf2	1.03E-173	-0.3646164	0.248	0.453	3.33E-169
6-Sep	3.31E-144	-0.3653306	0.47	0.681	1.07E-139

Nnat	3.71E-84	-0.3658345	0.811	0.901	1.20E-79
Cpe	4.92E-20	-0.3687787	0.684	0.656	1.59E-15
Pcp4	3.80E-63	-0.3698984	0.427	0.483	1.23E-58
Pcolce	4.86E-82	-0.3721437	0.411	0.564	1.57E-77
Traf4	2.19E-108	-0.3766567	0.621	0.734	7.07E-104
Eif5a	1.37E-199	-0.3771646	0.971	0.992	4.41E-195
Papss2	2.74E-68	-0.3798083	0.467	0.554	8.84E-64
Igfbp3	4.93E-90	-0.3800064	0.252	0.389	1.59E-85
Pfn1	1.08E-219	-0.3802532	0.967	0.992	3.47E-215
Fdps	2.60E-100	-0.3823796	0.646	0.783	8.39E-96
Tnc	4.54E-138	-0.3843248	0.246	0.466	1.47E-133
Dpysl3	0	-0.3845314	0.332	0.578	0
Jdp2	5.08E-139	-0.384992	0.222	0.41	1.64E-134
Basp1	3.21E-33	-0.3880366	0.819	0.911	1.03E-28
Mest	1.69E-161	-0.3890108	0.955	0.984	5.45E-157
Amot	4.10E-113	-0.3935619	0.443	0.585	1.32E-108
Selenom	4.69E-141	-0.3957764	0.658	0.822	1.51E-136
Cd24a	4.60E-88	-0.3959531	0.891	0.916	1.49E-83
Cnn2	1.11E-216	-0.3960035	0.484	0.703	3.58E-212
Ssbp2	4.91E-101	-0.3991874	0.671	0.793	1.58E-96
Bambi	4.20E-114	-0.4002117	0.377	0.54	1.35E-109
Maf	1.42E-262	-0.4008044	0.237	0.482	4.59E-258
Id2	4.18E-93	-0.402711	0.705	0.791	1.35E-88
Rflnb	1.26E-155	-0.4030144	0.478	0.631	4.08E-151
Hic1	2.46E-91	-0.412602	0.357	0.485	7.95E-87
Dlx5	9.12E-291	-0.4159224	0.348	0.531	2.94E-286
Meox1	1.19E-226	-0.4181753	0.263	0.483	3.85E-222
Gpc3	1.39E-181	-0.4208471	0.96	0.986	4.49E-177
Fkbp11	5.87E-105	-0.4210174	0.372	0.532	1.89E-100
Smarca2	5.51E-78	-0.422903	0.431	0.593	1.78E-73
Col9a2	3.00E-27	-0.4246079	0.812	0.725	9.69E-23
Tspan4	8.41E-99	-0.4266427	0.366	0.513	2.71E-94
Mia	4.00E-31	-0.4272175	0.776	0.695	1.29E-26
Tubb2b	1.20E-38	-0.4295724	0.451	0.533	3.89E-34
Pcolce2	5.82E-24	-0.4353978	0.272	0.356	1.88E-19
S100a6	3.70E-84	-0.4364806	0.136	0.314	1.19E-79
Srm	5.78E-136	-0.4366565	0.603	0.747	1.87E-131
Fxyd3	8.18E-30	-0.4503638	0.281	0.359	2.64E-25
Selenos	4.87E-170	-0.452114	0.627	0.796	1.57E-165
Ss18l2	6.82E-105	-0.4554734	0.548	0.654	2.20E-100
Pth1r	3.91E-140	-0.4679022	0.505	0.648	1.26E-135

Rtl3	2.63E-206	-0.4688834	0.181	0.428	8.49E-202
Limch1	2.59E-224	-0.4855585	0.344	0.59	8.36E-220
Cmtm5	1.13E-60	-0.4898329	0.246	0.355	3.63E-56
Tbrg1	7.61E-164	-0.4942996	0.74	0.848	2.46E-159
Serpinh1	6.02E-283	-0.5042257	0.966	0.988	1.94E-278
Fgfr3	1.24E-107	-0.5068804	0.296	0.43	4.01E-103
Grb10	9.09E-260	-0.5190929	0.866	0.945	2.94E-255
Nrp2	1.06E-250	-0.52668	0.34	0.586	3.43E-246
Susd5	9.42E-184	-0.5281288	0.141	0.369	3.04E-179
Flnb	4.78E-164	-0.5413567	0.485	0.64	1.54E-159
Comp	2.25E-61	-0.5413584	0.161	0.295	7.27E-57
Ihh	0	-0.5459183	0.159	0.474	0
Asb4	3.86E-148	-0.5482577	0.476	0.612	1.25E-143
Rspo3	6.57E-112	-0.5511871	0.318	0.471	2.12E-107
Id3	1.36E-213	-0.5574703	0.877	0.935	4.40E-209
Shox2	3.39E-224	-0.5583722	0.66	0.824	1.09E-219
Arpc1b	1.03E-222	-0.5640722	0.41	0.659	3.31E-218
Thbs1	2.20E-104	-0.5709692	0.371	0.498	7.11E-100
Hes1	4.58E-159	-0.5767231	0.406	0.607	1.48E-154
Pim3	1.35E-157	-0.5906678	0.488	0.637	4.35E-153
Peg10	8.80E-117	-0.5917444	0.446	0.567	2.84E-112
Cthrc1	2.08E-155	-0.6045431	0.845	0.865	6.72E-151
Lgals3	3.69E-94	-0.6189701	0.17	0.324	1.19E-89
Col1a2	1.30E-160	-0.6336426	0.682	0.851	4.19E-156
Vim	2.31E-136	-0.6344528	0.812	0.892	7.45E-132
Runx3	0	-0.6350026	0.261	0.569	0
Islr	2.49E-264	-0.6383287	0.47	0.693	8.05E-260
Twist2	0	-0.6448197	0.484	0.665	0
Ugdh	1.00E-151	-0.6450741	0.575	0.678	3.23E-147
Id1	2.30E-203	-0.6641462	0.797	0.881	7.42E-199
Ptch1	0	-0.6938226	0.547	0.76	0
Stk26	2.66E-227	-0.7210497	0.468	0.636	8.59E-223
Ebf1	0	-0.726277	0.43	0.73	0
Runx2	0	-0.7445826	0.393	0.629	0
Lbhd2	3.27E-212	-0.7465417	0.195	0.429	1.06E-207
Prrx1	1.46E-242	-0.7532261	0.535	0.796	4.71E-238
Phlda2	2.92E-86	-0.7540517	0.402	0.498	9.43E-82
Cnmd	9.10E-202	-0.7607021	0.397	0.554	2.94E-197
Crabp1	3.64E-107	-0.7796922	0.583	0.705	1.18E-102
Snai1	0	-0.8061094	0.498	0.686	0
Gdf10	0	-0.8191565	0.164	0.489	0

Col11a2	1.88E-194	-0.8226674	0.427	0.569	6.07E-190
Col9a3	2.50E-83	-0.8425244	0.848	0.733	8.08E-79
Acan	6.00E-211	-0.8649823	0.401	0.555	1.94E-206
Mef2c	0	-0.8806299	0.277	0.554	0
Dcn	9.18E-12	-0.8869695	0.324	0.325	2.96E-07
Lum	9.82E-123	-0.9539748	0.324	0.485	3.17E-118
Wwp2	1.26E-129	-0.988376	0.729	0.709	4.07E-125
Snorc	5.61E-114	-1.0986529	0.203	0.366	1.81E-109
Col11a1	9.88E-238	-1.1930335	0.732	0.769	3.19E-233
Hmga2	0	-1.223218	0.671	0.941	0
Cytl1	0	-1.2258278	0.197	0.504	0
Col2a1	1.67E-139	-1.2708661	0.934	0.801	5.38E-135
Col9a1	0	-1.4532065	0.521	0.683	0
Hapln1	1.80E-301	-1.5092767	0.259	0.493	5.83E-297
Matn4	0	-1.6939288	0.627	0.749	0
Matn1	0	-2.0946313	0.237	0.542	0

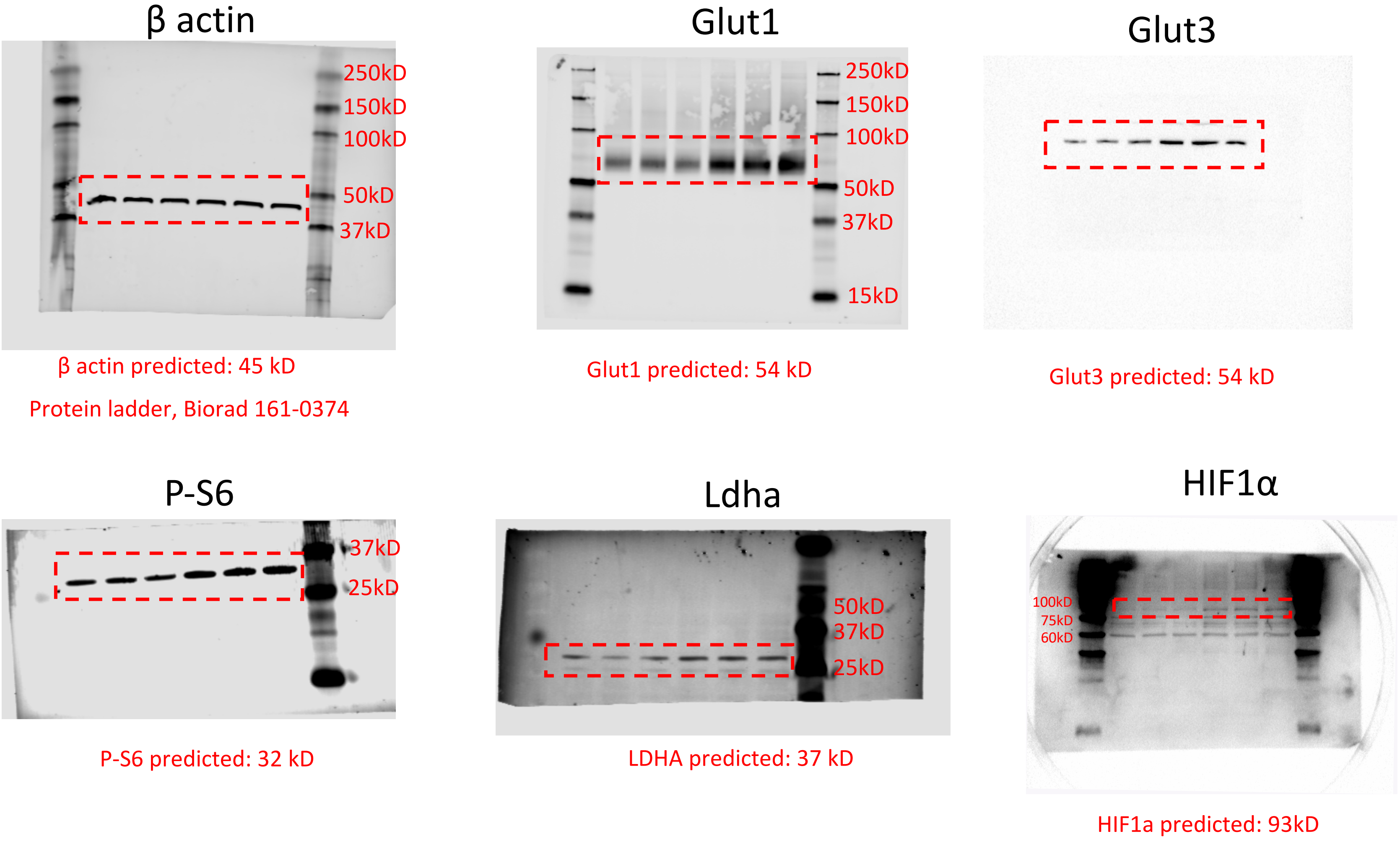
β actin
250kD
150kD
100kD
50kD
37kD
β actin predicted: 45 kD
Protein ladder, Biorad 161-0374
Glut1
250kD
150kD
100kD
50kD
37kD
15kD
Glut1 predicted: 54 kD
Glut3
Glut3 predicted: 54 kD
P-S6
37kD
25kD
P-S6 predicted: 32 kD
Ldha
50kD
37kD
25kD
LDHA predicted: 37 kD
HIF1α
100kD
75kD
60kD
HIF1a predicted: 93kD

Figure 8A

Smad2 β actin

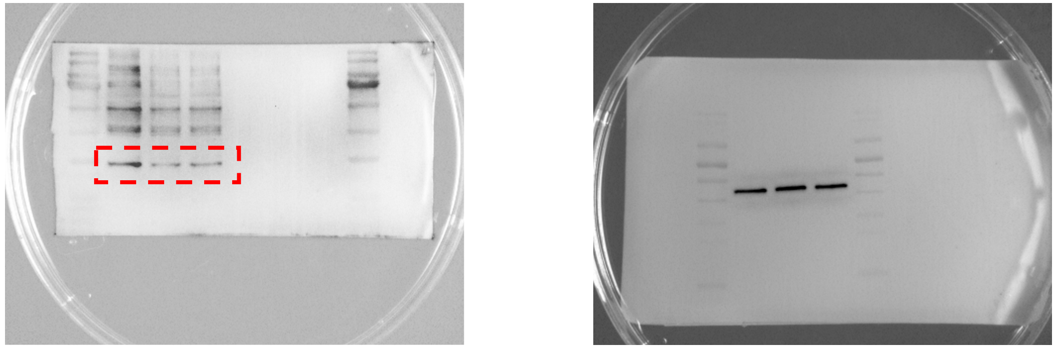

Figure 9A

Glut1 β actin

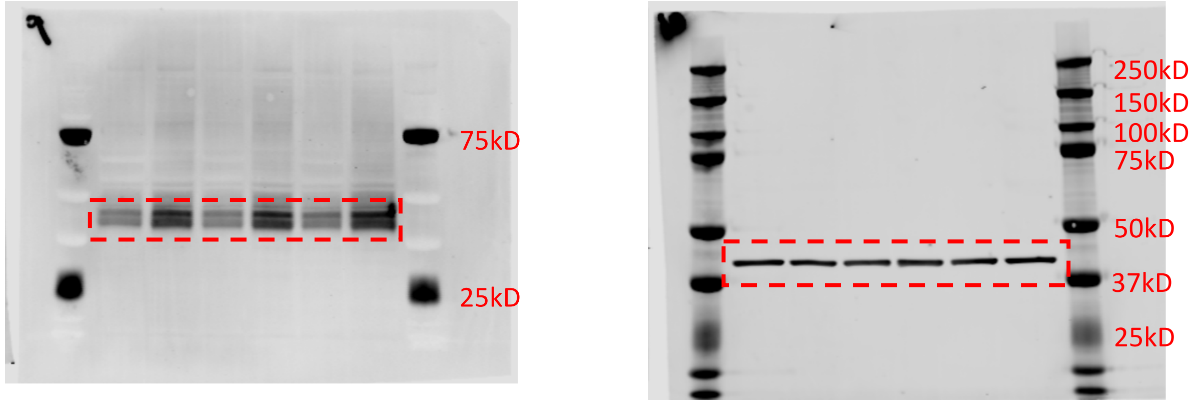

Figure 9D

β actin and P-S6

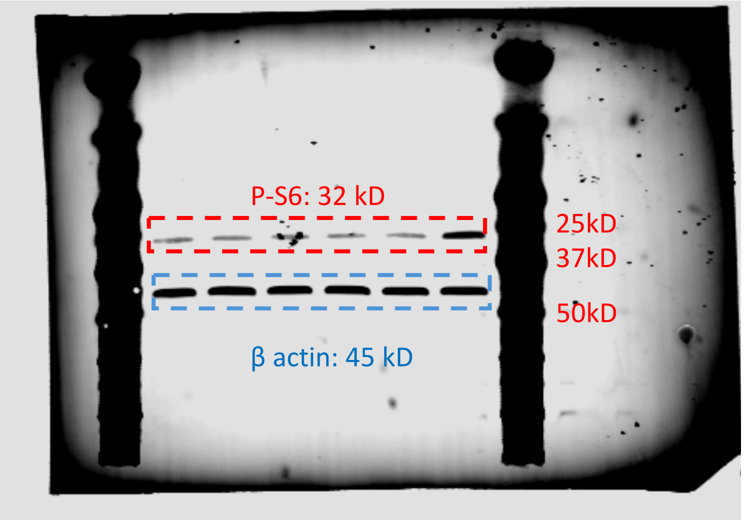

P-S6K

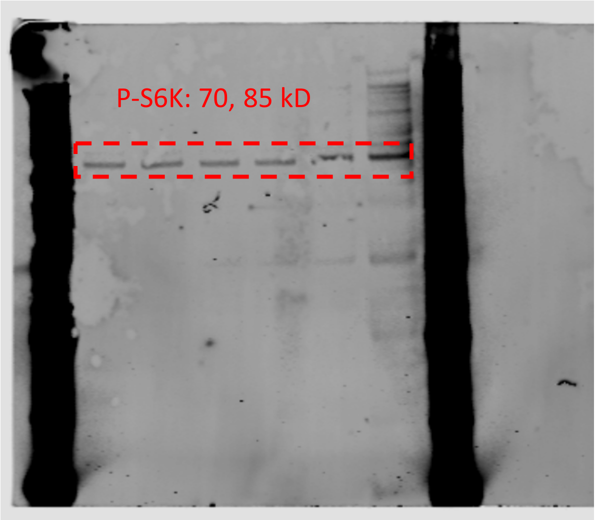

Smad2

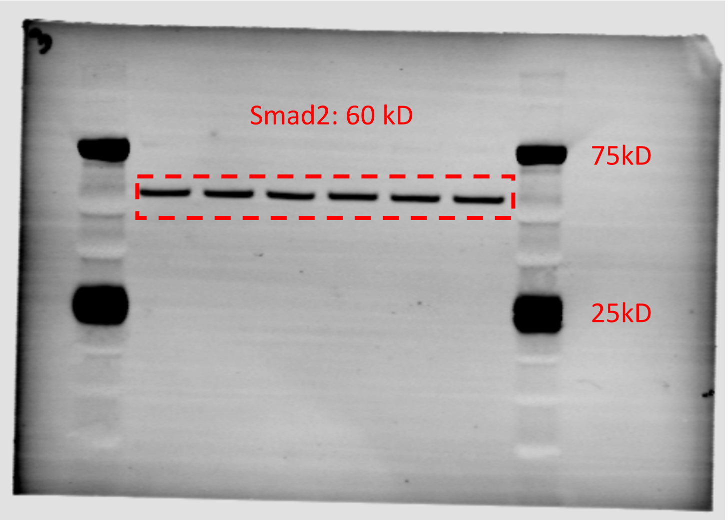

P-Smad2

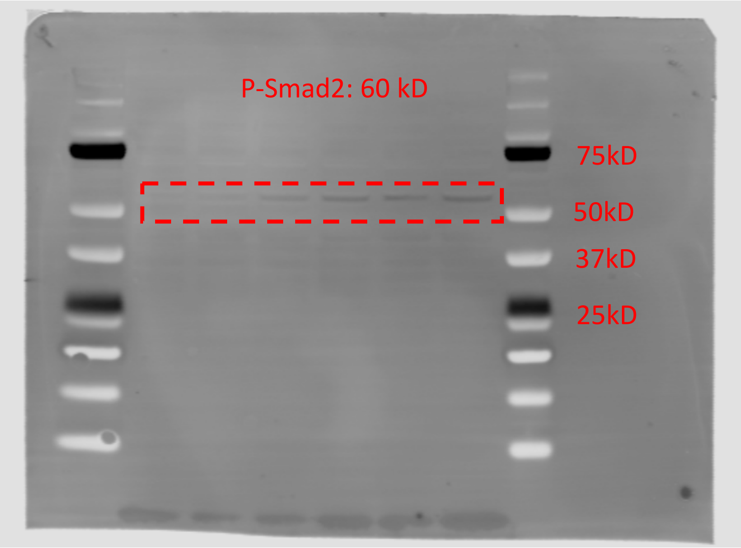

S6

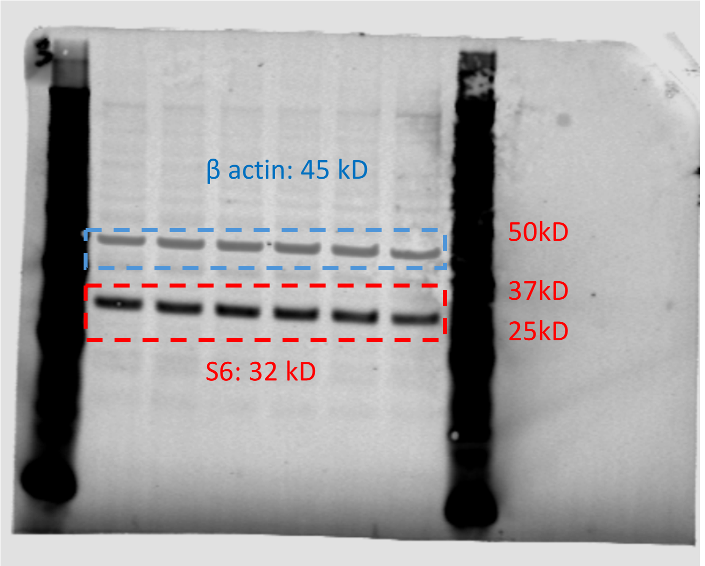

Figure 9E
Images shown in paper

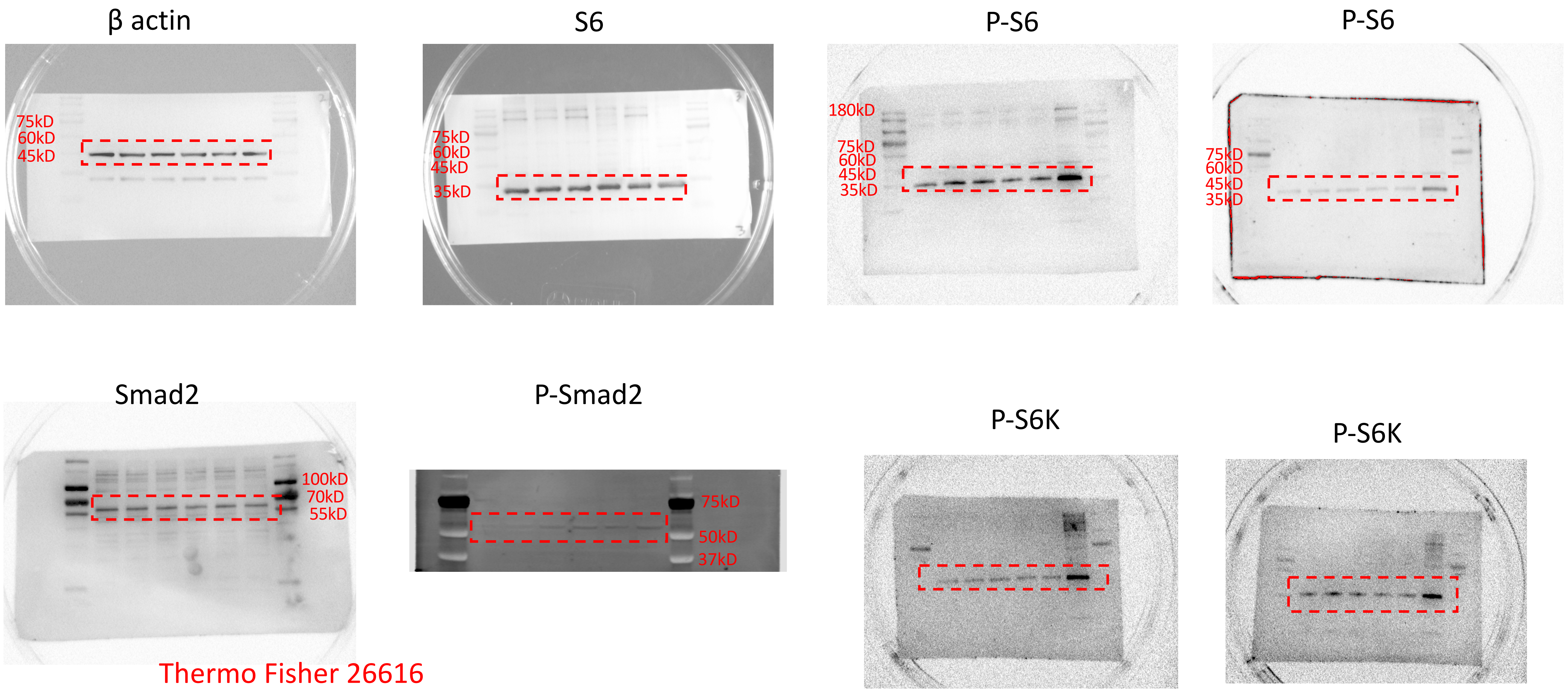

Fig. 9E

Additional images used for quantification

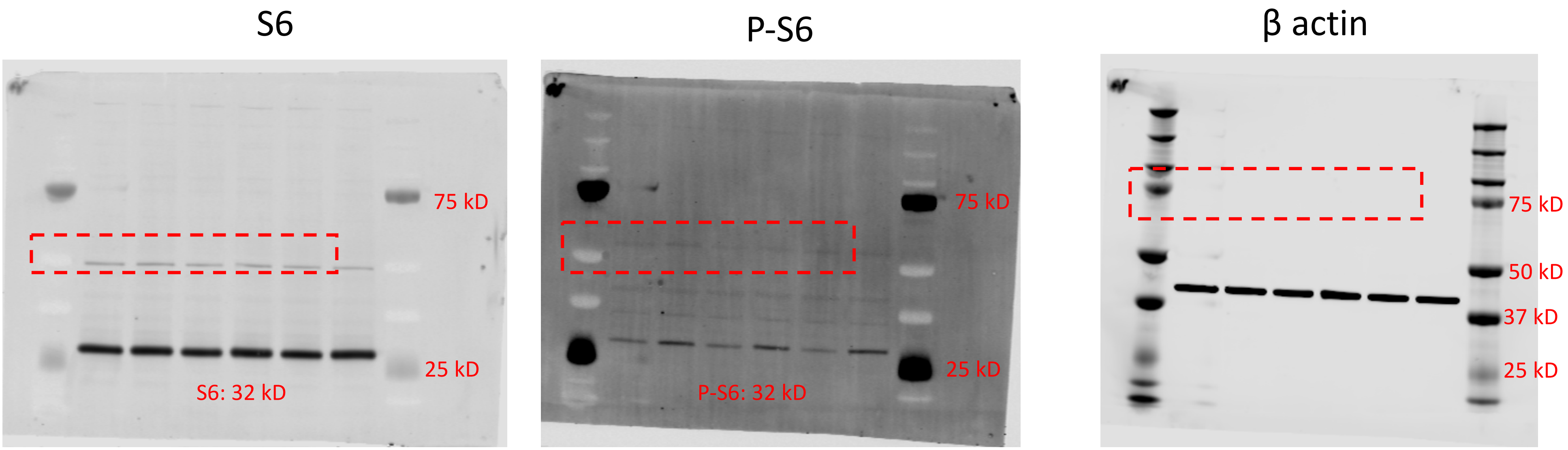
S6
75 kD
25 kD
S6: 32 kD
P-S6
75 kD
25 kD
P-S6: 32 kD
β actin
75 kD
50 kD
37 kD
25 kD

Figure 9F

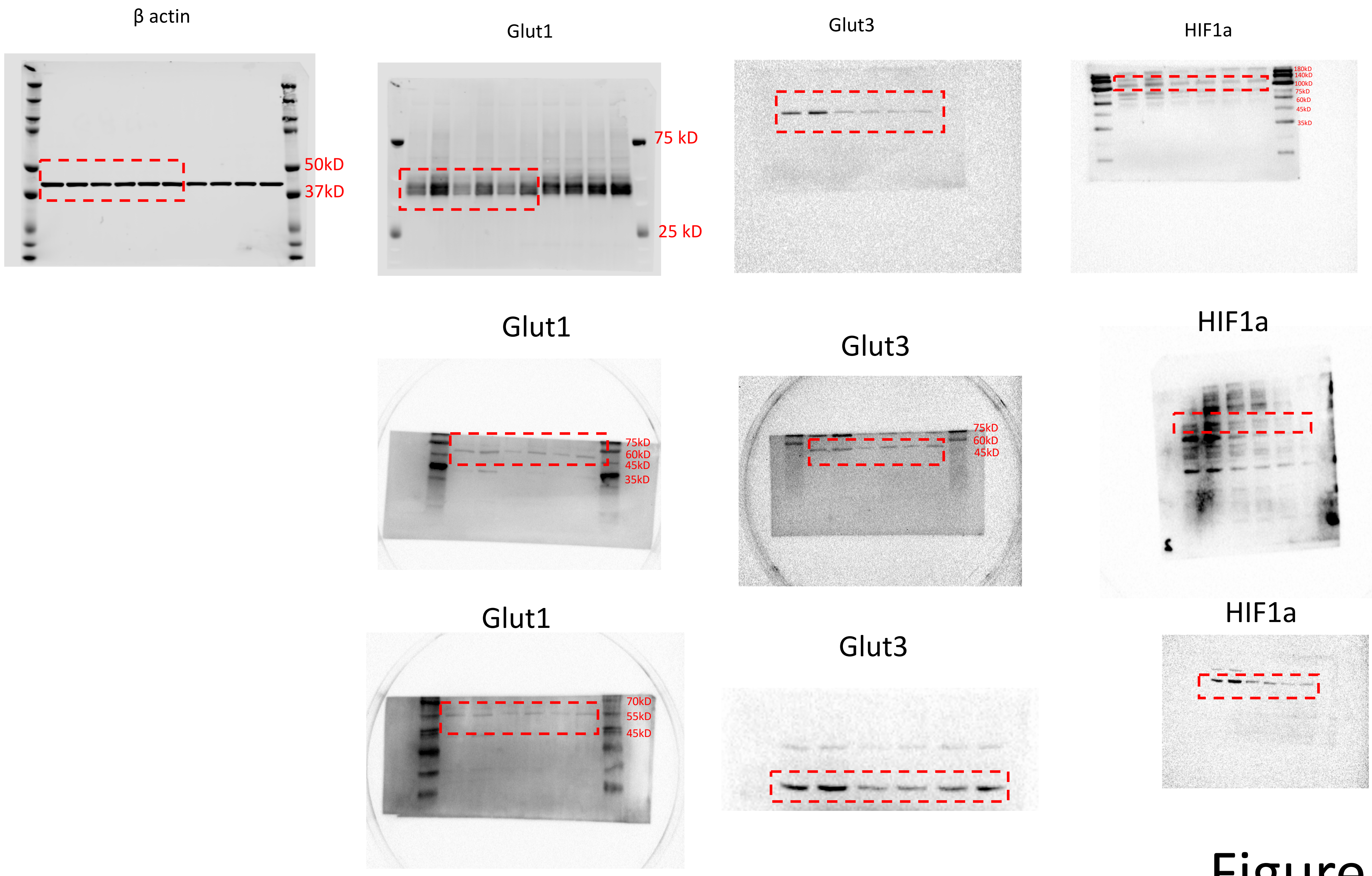

Images shown in paper

Figure 10A

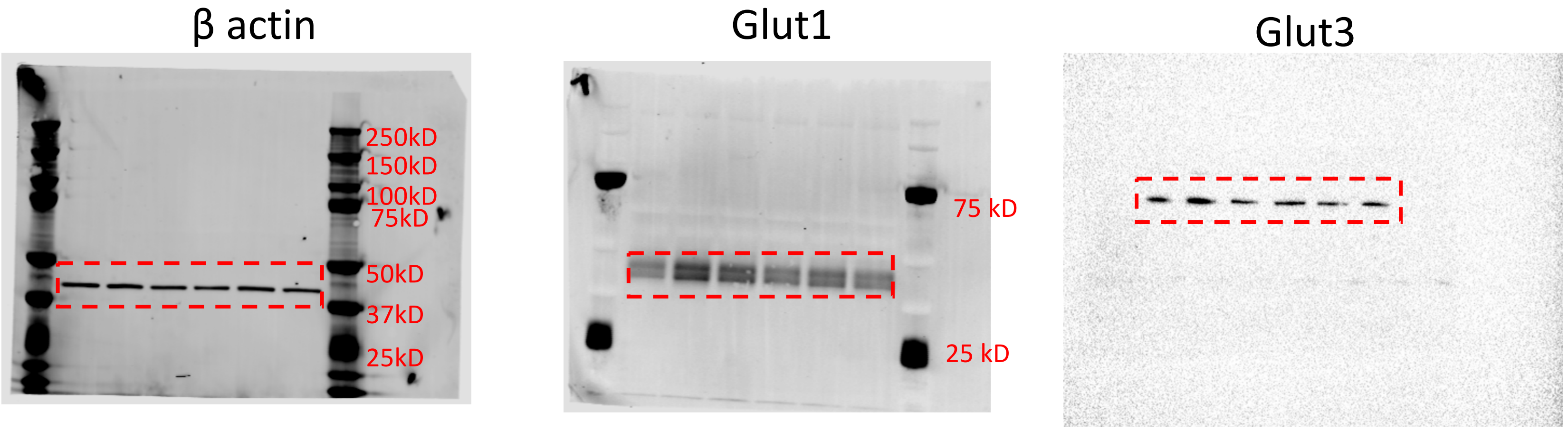
β actin
250kD
150kD
100kD
75kD
50kD
37kD
25kD
Glut1
75 kD
25 kD
Glut3

Figure 10D

Glut1

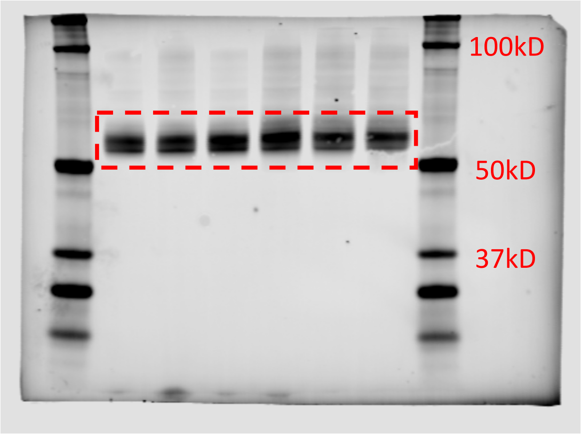

Glut1 predicted: 54 kD

Glut3

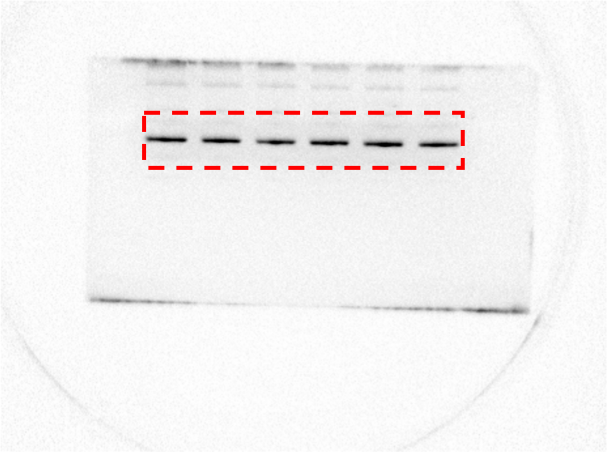

Glut3 predicted: 54 kD

HIF1α

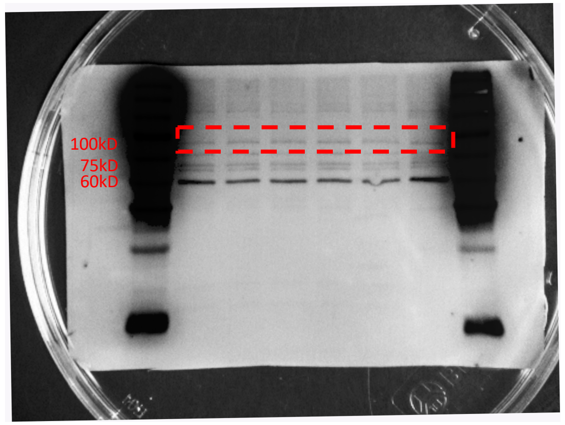

HIF1a predicted: 93kD

8h Ldha

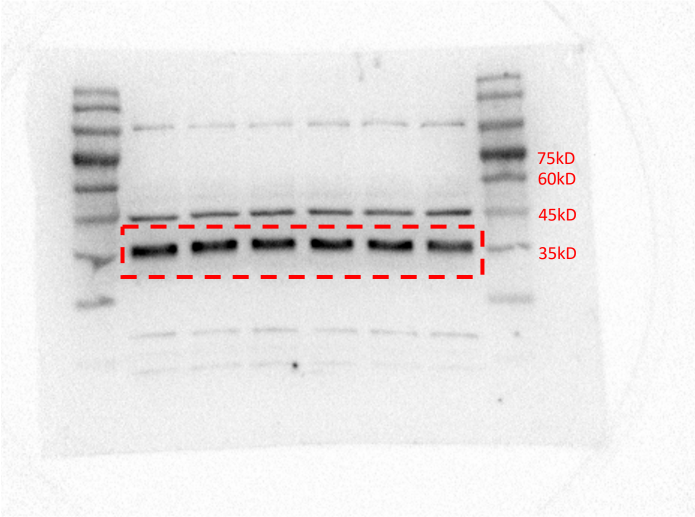

LDHA: 37 kD

Figure S7A

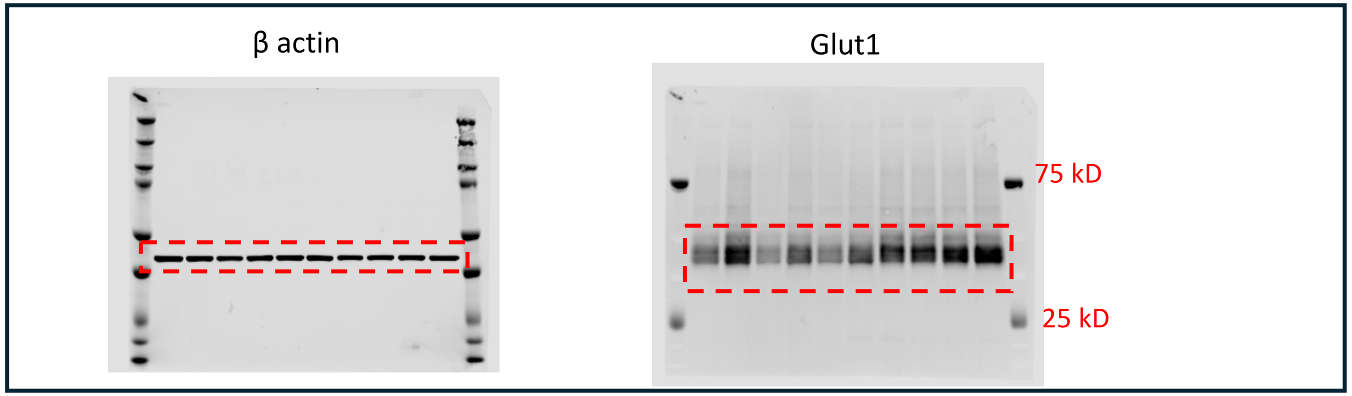

Figure S8D

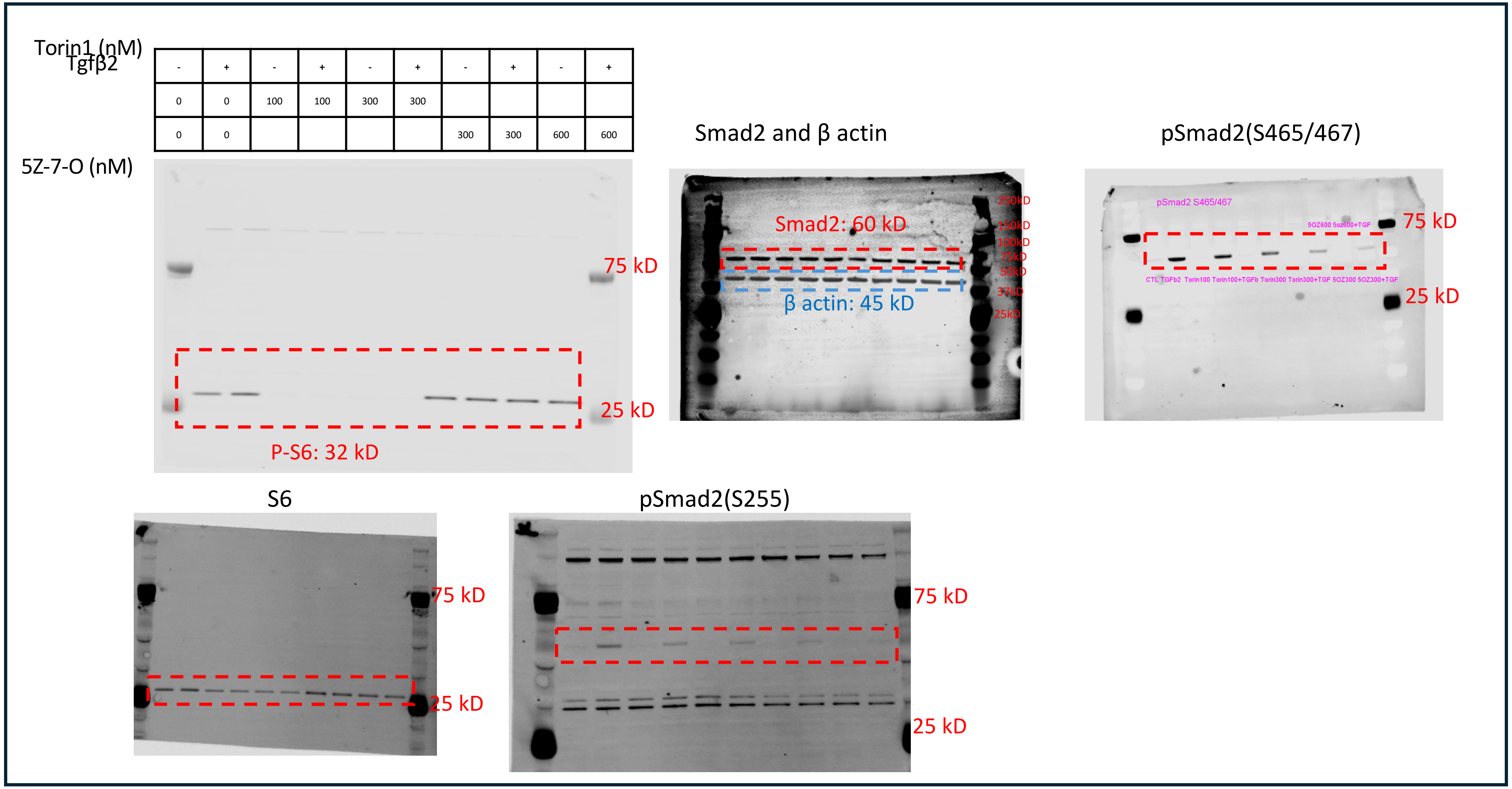

Figure S8E
